# Harnessing microbial consortia for induced systemic resistance and sustainable management of dry root rot in cluster bean under hot arid climatic conditions

**DOI:** 10.3389/fmicb.2025.1699101

**Published:** 2025-10-22

**Authors:** Devendra Singh, Aman Verma, Kuldeep Singh Jadon, Hans Raj Mahla, Rajesh Kumar Kakani

**Affiliations:** Division of Plant Improvement and Pest Management, ICAR-Central Arid Zone Research Institute, Jodhpur, India

**Keywords:** biocontrol, biochemical modulation, induced resistance, plant defense enzyme, sustainable agriculture

## Abstract

Dry root rot, caused by *Macrophomina phaseolina*, severely threatens cluster bean production, necessitating sustainable management strategies. This study aimed to screen, characterize and evaluate microbial isolates with antagonistic potential against *M. phaseolina*. Among 763 isolates, *Trichoderma breve* 37F, *Pseudomonas* sp. 8B, *Aneurinibacillus aneurinilyticus* 16B, and *Bacillus velezensis* 32B exhibited strong biocontrol and plant growth-promoting traits. All four biocontrol agents demonstrated good compatibility. Pot experiments revealed that the four-microbe consortium comprising *T. breve* 37F + *Pseudomonas* sp. 8B + *A. aneurinilyticus* 16B + *B. velezensis* 32B significantly suppressed *M. phaseolina*, achieving 87.13% disease control and declining the percent disease incidence (PDI) to 16.7%. This consortium also enhanced plant growth, increasing plant height (1.66-fold), fresh weight (2.81-fold), dry weight (2.56-fold) and yield (21.4-fold) over the infected control. Significant improvements were observed in plant physiological and biochemical attributes, including increased total chlorophyll (3.16-fold), carotenoids (1.95-fold), total phenols (2.11-fold), flavonoids (2.53-fold), antioxidant activity (3.21-fold) and tannins (4.72-fold), alongside a 46.8% reduction in electrolyte leakage. Antioxidant enzyme activities, including peroxidase (4.05-fold), polyphenol oxidase (2.69-fold), phenylalanine ammonia lyase (1.93-fold), tyrosine ammonia lyase (2.01-fold), superoxide dismutase (2.94-fold) and catalase (2.25-fold), were significantly upregulated in consortium-treated plants. Field validation confirmed the efficacy of the four-microbe consortium, reducing PDI to 40.0% (42.0% disease control) while enhancing seed yield by 2.79-fold and 1.67-fold over the infected and mock controls, respectively. These findings demonstrate the potential of a microbial consortium as an eco-friendly biocontrol strategy. Future work should focus on formulation and large-scale field validation.

## 1 Introduction

Cluster bean (*Cyamopsis tetragonoloba* L.), a drought-tolerant legume primarily cultivated in arid and semi-arid regions, plays a significant role in food, fodder and industrial applications, particularly in guar gum production (Mahla et al., 2024). However, its productivity is severely impacted by various biotic and abiotic stresses, among which dry root rot caused by *M. phaseolina* is one of the most devastating soil-borne diseases. This pathogen has a broad host range and thrives under high temperatures and low soil moisture conditions, leading to substantial yield losses (Alizadeh et al., 2025). The disease symptoms include root necrosis, vascular discoloration, and plant wilting, ultimately resulting in plant death. The persistent nature of *M. phaseolina* through microsclerotia in soil further complicates its management, necessitating effective and sustainable control strategies (Basandrai et al., 2021).

Traditionally, chemical fungicides such as carbendazim, thiophanate-methyl, and tebuconazole have been widely used to control *M. phaseolina* (Singh D. et al., 2024; Singh M. et al., 2024). While these chemicals offer immediate disease suppression, their prolonged application poses significant environmental and health hazards, including soil toxicity, disruption of beneficial microbial communities and the emergence of fungicide-resistant pathogen strains (Zhang et al., 2024). Moreover, chemical control methods are often unsustainable in arid regions due to rapid degradation under extreme climatic conditions. These limitations have necessitated the exploration of alternative eco-friendly approaches, particularly biological control methods, which leverage antagonistic microorganisms to suppress soil-borne pathogens. Moreover, biofungicides are less likely to contribute to resistance development in pathogens, enhancing their long-term efficacy (Fenta and Mekonnen, 2024).

Microbial biocontrol agents (BCAs) such as *Trichoderma* spp., *Pseudomonas fluorescens*, *Bacillus* spp. and *Aneurinibacillus* spp. have shown promising potential against *M. phaseolina* through various mechanisms, including mycoparasitism, antibiotic production, competition for nutrients and induction of systemic resistance in plants (Rangel-Montoya et al., 2022; Rubayet and Bhuiyan, 2023). Mycoparasitism involves the secretion of cell wall-degrading enzymes (chitinases, glucanases etc.) which play a crucial role in breaking down the structural components of pathogenic fungi (Marquez et al., 2021). However, individual biocontrol agents often exhibit inconsistent efficacy under field conditions due to environmental variability and pathogen adaptability. In contrast, microbial consortia, comprising multiple compatible biocontrol strains, have demonstrated superior disease suppression and plant growth promotion through synergistic interactions (Kumar et al., 2023; Negi et al., 2024). Such consortia offer multiple modes of action, ensuring robustness and resilience against environmental fluctuations, making them a more viable and sustainable alternative to chemical fungicides (Vishwakarma et al., 2020; Poveda and Eugui, 2022).

Despite the growing evidence supporting the efficacy of microbial consortia, research gaps remain in their systematic evaluation, optimization and field validation under arid climatic conditions. This study hypothesizes that a well-characterized microbial consortium comprising compatible BCAs can provide enhanced biocontrol efficacy against *M. phaseolina* compared to individual strains. By integrating *in vitro* screening, pot trials and field validation, this research aims to bridge the knowledge gap and establish a reliable, eco-friendly strategy for dry root rot management in cluster bean. Unlike previous studies, which primarily focus on single-strain efficacy, this work emphasizes the collective impact of multiple biocontrol agents in improving plant health and productivity in arid regions.

The major objectives of this study are to isolate and characterize microbial strains with biocontrol potential against *M. phaseolina*, assess their compatibility to develop an effective microbial consortium and evaluate the consortium’s efficacy in suppressing dry root rot through pot and field trials. Additionally, the study aims to analyze plant growth-promoting traits and the induction of host defense responses while validating the microbial consortium as a sustainable disease management strategy in arid agro-ecosystems.

## 2 Materials and methods

### 2.1 Dual plate assay

A total of 480 bacterial and 283 fungal isolates, obtained from our previous study (Singh D. et al., 2024; Singh M. et al., 2024), were screened for antagonistic activity against *M. phaseolina* (ITCC Accession No-7863), obtained from the Indian Type Culture Collection, IARI, New Delhi. The screening was performed using the dual culture technique on Potato Dextrose Agar (PDA) in triplicate. For fungal isolates, an actively growing culture of each test isolate and *M. phaseolina* were placed at equal distances from the center of a PDA plate. In the case of bacterial isolates, a 24 h old bacterial culture was streaked parallel to *M. phaseolina* at equal distances on opposite sides of the plate. The plates were incubated at 28 ± 2 °C for 5 days. Control plates contained only *M. phaseolina*. The antagonistic potential of each isolate was evaluated by measuring the mycelial growth inhibition of *M. phaseolina* on test plates compared to the control. The percentage inhibition was determined using the following Equation 1 (El-Sayed et al., 2014):


(1) $$\text{Percentage of Inhibition}=\frac{\left(\begin{array}{l} \text{Control Mycelial Diameter}\\ -\text{Test Mycelial Diameter} \end{array}\right)}{\text{Control Mycelial Diameter}\;}*100$$

### 2.2 Microbial traits for plant growth promotion and disease suppression

The selected isolates were assessed for their biocontrol potential and plant growth-promoting traits. Siderophore production was examined by spot inoculating the isolates onto nutrient agar supplemented with chrome azurol S (CAS) dye solution, as described by Milagres et al. (1999). HCN production was determined using Castric’s method (Castric, 1975), while ammonia production was detected following Dye’s method (Dye, 1962). Hydrolytic enzyme activities, including chitinase, chitosanase and β-1,3-glucanase, were evaluated using a well diffusion assay with specific substrates-chitin for chitinase, laminarin for β-1,3-glucanase and chitosan for chitosanase (Zou et al., 2002; Zacky and Ting, 2013; Saito et al., 2009). Clear zones were recorded after 24 h at 37 °C. Phosphorus solubilization was evaluated using Pikovskaya’s agar medium (Pikovskaya, 1948), while zinc solubilization was evaluated using Tris-minimal salt medium supplemented with 1% ZnO (Fasim et al., 2002). IAA production was quantified using Patten and Glick’s method (Patten and Glick, 2002).

### 2.3 *In vitro* compatibility

The *in vitro* compatibility of *T. breve* 37F with bacterial isolates (8B, 16B and 32B) was evaluated using a cross-streak quadrant method on nutrient agar (NA) plates. The plates were divided into four quadrants by marking perpendicular lines on the back of each plate. Three bacterial isolates were streaked separately in three quadrants using a sterile inoculating loop, while a 9 mm mycelial disk of *T. breve* 37F from an actively growing culture was placed in the fourth quadrant. The streaks were positioned approximately 1 cm away from each other to allow observation of potential interactions. The plates were first incubated at 30 °C for 24 h to facilitate bacterial growth. After bacterial establishment, the plates were further incubated at 28 ± 2 °C for 5–7 days to allow fungal growth and interaction assessment. Compatibility was determined based on growth patterns, with no inhibition or overgrowth indicating a positive interaction, while the presence of inhibition zones suggested antagonism (Rajeela et al., 2018).

### 2.4 Characterization of selected biocontrol agents: morphological, physiological and biochemical aspects

Bacterial isolates were characterized based on their morphological, physiological and biochemical properties. Morphological characterization included an assessment of colony morphology, Gram staining and the KOH solubility test. Physiological and biochemical characterization involved evaluating the utilization of 33 different carbon sources, along with amino acid metabolism and enzymatic activities. Additionally, H₂S production ability was assessed. Biochemical assays were performed using the HiCarbo™ Kit (KB 009) and HiAssorted™ Biochemical Test Kit (KB002). Each well in the kits was inoculated with 50 μL of bacterial culture, adjusted to an optical density (OD) of 0.5 at 620 nm, and incubated at 35–37 °C for 24–48 h. Observations were recorded based on color changes. For fungal isolates, microscopic examination was conducted to assess key morphological traits.

### 2.5 Molecular identification of selected isolates using 16S rRNA and ITS regions

Genomic DNA from bacterial and fungal isolates was extracted using ZYMO Research DNA isolation kits (United States), following the manufacturer’s protocols for bacteria and fungi separately. The quality and concentration of DNA were assessed using a NanoDrop spectrophotometer (Thermo Scientific) and integrity was confirmed via 0.8% agarose gel electrophoresis. The 16S rRNA gene was amplified using universal primers PA (5′-AGAGTTTGATCCTGGCTCAG-3′) and PH (5′-AAGGAGGTGATCCAGCCGCA-3′) (Edwards et al., 1989). The ITS region was amplified with primers ITS-1 (5′-TCCGTAGGTGAACCTGCGG-3′) and ITS-4 (5′-TCCTCCGCTTATTGATATGC-3′) (White et al., 1990). PCR reactions (25.0 μL) contained 12.5 μL Taq PCR Master Mix (Thermo Scientific), 1.0 μL of each primer (10.0 μM), 1.0 μL genomic DNA (~50.0 ng) and 9.5 μL nuclease-free water. Amplification conditions included an initial denaturation at 94 °C for 5 min, followed by 35 cycles of denaturation (94 °C, 30 s), annealing (55 °C for 16S rRNA; 52 °C for ITS, 30 s), extension (72 °C, 1 min), and a final extension at 72 °C for 10 min. PCR products were visualized on a 1.2% agarose gel stained with ethidium bromide, purified using a Qiagen PCR purification kit (Germany) and sequenced using the Sanger method (Centyle Biotech Pvt. Ltd., New Delhi). Sequences were analyzed using BLASTn against the NCBI GenBank database (Altschul et al., 1990). Phylogenetic relationships were inferred using the Maximum Likelihood method (Tamura and Nei, 1993) in MEGA software (Kumar et al., 2018), with evolutionary distances estimated using the Kimura 2-parameter model (Kimura, 1980). The sequences of were submitted to NCBI GenBank, with accession numbers OR105515, PP869086, PP064118 and PP863874 for isolates 37F, 8B, 16B and 32B, respectively.

### 2.6 Pot experiment

A pot experiment was conducted in a net house at ICAR-CAZRI, Jodhpur, to evaluate the potential of biocontrol agents, against *M. phaseolina* in cluster bean (Supplementary Figure S1). The study assessed disease suppression, plant growth parameters and induced systemic resistance (ISR). Pots (25 cm × 25 cm) were filled with 10 kg of a sterilized sand–soil mixture (1:3) prepared by tyndallization. Seeds of the susceptible variety Pusa Navbahar were surface-sterilized with 70% ethanol for 1 min, followed by 1.5% sodium hypochlorite for 5 min (Minchev et al., 2021), and then treated with bacterial isolates (8B, 16B, 32B) and the fungal isolate (37F). Bacterial cultures were grown in nutrient broth at 30 °C for 24 h, while *Trichoderma* was cultivated in potato dextrose broth for 7 days. The final concentrations were standardized to 10^8^ CFU mL^−1^ for bacteria and 10^4^ spores mL^−1^ for *Trichoderma*. These prepared suspensions were then mixed in equal proportions to formulate the microbial consortia, following the treatment combinations specified in the experimental design. Treated seeds were coated with biocontrol agents using charcoal as a carrier and air-dried before sowing. Seeds treated with sterile water and charcoal served as controls. The experiment included 17 treatments in a Completely Randomized Design (CRD) with six replications (Table 1). A talc-based formulation of biocontrol agents was applied at 10 g/kg of soil at 20, 30 and 40 days after sowing (DAS). The pathogen *M. phaseolina* was introduced through drenching at 50 DAS using sorghum-based inoculum (10^4^ spores g/soil) (Irulappan et al., 2021). Fertilization was carried out using the recommended doses (RDF), applying 20 kg N/ha and 40 kg P₂O₅/ha, as per Singh (2014). Following germination, six plants were maintained per pot. Growth parameters (plant height, biomass, number of pods per plant and seed yield) were recorded, with three replications used for disease assessment and yield, while the other three were used for biochemical analyses.

**Table 1:** Evaluation of bioefficacy of selected biocontrol agents in individual and consortium mode against *Macrophomina phaseolina* of cluster bean in pot experiment during 2023.

Treatments	PDI (percent disease incidence)	Percent disease control over the positive control
T1: No pathogen + No biocontrol agent (mock control)	12.5 ± 7.2^f^	–
T2: Only *Macrophomina phaseolina* (infected control)	91.7 ± 14.4^a^	–
T3: *Trichoderma breve* 37F + challenged with *M. phaseolina*	41.7 ± 8.3^bcdef^	54.5
T4: *Pseudomonas* sp. 8B + challenged with *M. phaseolina*	62.5 ± 7.2^ab^	31.8
T5: *Aneurinibacillus aneurinilyticus* 16B + challenged with *M. phaseolina*	62.5 ± 7.2^ab^	31.8
T6: *Bacillus velezensis* 32B + challenged with *M. phaseolina*	54.2 ± 13.8^bcd^	40.9
T7: 37F + 8B + challenged with *M. phaseolina*	33.3 ± 11.8^bcdef^	63.7
T8: 37F + 16B + challenged with *M. phaseolina*	29.2 ± 7.2^cdef^	68.2
T9: 37F + 32B + challenged with *M. phaseolina*	29.2 ± 7.2^cdef^	68.2
T10: 8B + 16B + challenged with *M. phaseolina*	58.3 ± 8.3^bc^	36.4
T11: 8B + 32B + challenged with *M. phaseolina*	54.2 ± 7.2b^cd^	40.9
T12: 16B + 32B + challenged with *M. phaseolina*	50.0 ± 11.8^bcde^	45.5
T13: 37F + 8B + 16B + challenged with *M. phaseolina*	25.0 ± 8.3^def^	72.7
T14: 37F + 8B + 32B + challenged with *M. phaseolina*	25.0 ± 8.3^def^	72.7
T15: 37F + 16B + 32B + challenged with *M. phaseolina*	20.8 ± 13.8^ef^	77.3
T16: 8B + 16B + 32B + challenged with *M. phaseolina*	41.7 ± 8.3^bcdef^	54.5
T17: 37F + 8B + 16B + 32B + challenged with *M. phaseolina*	16.7 ± 11.8^f^	87.13

*Data are the average of three replicates ± SD; Grouping information between mean values of obtained data was carried out by Fisher LSD Method and 95% confidence (p ≤ 0.05). Different letter point out significant differences in a column.

#### 2.6.1 Percent disease incidence

Percent disease incidence (PDI) was assessed to evaluate the effectiveness of biocontrol agents in managing *M. phaseolina* in cluster bean. Disease symptoms were observed at regular intervals and PDI (Equation 2) and Percent Disease Control (PDC) (Equation 3) were calculated using the following formulas (Islam et al., 2018):


(2) $$\mathrm{PDI}=\frac{\text{Number of infected plants}}{\text{Total number of plants}\;}\times 100$$

(3) $$\mathrm{PDC}=\frac{\mathrm{PDI}\;\text{of control}­\mathrm{PDI}\;\text{of treatment}}{\mathrm{PDI}\;\text{of control}}\times 100$$

#### 2.6.2 Chlorophyll and carotenoids estimation

Chlorophyll content was estimated following the method described by Arnon (1949). Fresh leaf samples (0.1 g) were homogenized in 80% acetone and centrifuged at 10,000 rpm for 10 min. The absorbance of the supernatant was measured at 665, 647 and 461 nm using a spectrophotometer (Systronics, India) and total chlorophyll content was calculated using Arnon’s Equations 4–6.


(4) $$\text{Chlorophyll}\;a\;(\mathrm{mg}/g\;)=12.25\;(A665)-2.79\;(A647)*\frac{V}{1000*W\;}\;$$

(5) $$\text{Chlorophyll}\;b\;(\mathrm{mg}/g)=21.5(A647)-5.1\;(A665)*\frac{V}{1000*W}$$

(6) $$\text{Total Chlorophyll}\;(\mathrm{mg}/g)=7.15(A665)-18.71\;(A647)*\frac{V}{1000*W\;}$$

where:

A = absorbance at 663, 645 and 470 (nm); V = Final volume of chlorophyll extract in 80% acetone; W = Fresh weight (g).

Carotenoid content was determined following the protocol of Lichtenthaler and Wellburn (1983). The absorbance of the acetone-extracted pigment solution was recorded at 470 nm and the carotenoid concentration was calculated using standard Equation 7.


(7) $$\text{Carotenoid}\;(\mathrm{mg}/g)=((\frac{\begin{array}{l} 1000(A470)-(1.82*\mathrm{Chl}.a)\\ -(85.02*\mathrm{Chl}.b) \end{array}}{198})*\frac{V}{1000}*W)$$

#### 2.6.3 Estimation of total phenol

Total phenol content was estimated using the Folin–Ciocalteu method (Dewanto et al., 2002). Fresh leaf samples (0.5 g) were homogenized in 80% methanol and centrifuged at 10,000 rpm for 10 min. A 1 mL aliquot of the supernatant was mixed with 5 mL of Folin–Ciocalteu reagent (diluted 1:10) and incubated for 5 min, followed by the addition of 4 mL of 7% sodium carbonate solution. The mixture was incubated in the dark for 30 min, and absorbance was recorded at 765 nm. Total phenol content was expressed as mg gallic acid equivalent (GAE) per gram of fresh weight or (mg GAE/g F. W.).

#### 2.6.4 Estimation of flavonoid

A colorimetric method described by Zou et al. (2004) was used for estimation of Flavonoid content. A 1 mL aliquot of the methanolic extract was mixed with 4 mL of distilled water and 0.3 mL of 5% sodium nitrite solution, followed by incubation for 5 min. Then, 0.3 mL of 10% aluminum chloride solution was added and after another 5 min, 2 mL of 1 M sodium hydroxide was added to the reaction mixture. The final volume was adjusted to 10 mL with distilled water and absorbance was measured at 415 nm. Flavonoid content was quantified in terms of mg quercetin equivalent (QE) per gram of fresh weight (mg QE/g F. W.) for standardized expression.

#### 2.6.5 Estimation of antioxidant

The antioxidant activity of plant samples was estimated using the method described by Arnao et al. (2001). Fresh leaf samples (0.5 g) were homogenized in 80% methanol and centrifuged at 10,000 rpm for 10 min. The supernatant (100 μL) was mixed with 2 mL of 0.1 mM DPPH (2,2-diphenyl-1-picrylhydrazyl) solution prepared in methanol. The reaction mixture was incubated in the dark at room temperature for 30 min, and absorbance was recorded at 517 nm using a UV–Vis spectrophotometer (Systronics, India). A standard curve was prepared using Trolox, and antioxidant activity was expressed as Mm Trolox equivalents per gram of fresh weight (Mm Trolox/g F. W.).

#### 2.6.6 Estimation of tannin

Tannin content was quantified using the Vanillin-HCl method (Price and Scoyoc, 1978). Fresh leaf samples (0.5 g) were homogenized in 80% methanol and centrifuged at 10,000 rpm for 10 min. The supernatant (100 μL) was mixed with 1 mL of 4% vanillin in methanol and 1.5 mL of concentrated HCl. The reaction mixture was incubated at room temperature for 20 min, and absorbance was measured at 500 nm using a UV–Vis spectrophotometer (Systronics, India). A calibration curve was prepared using catechin (0–100 μg/mL) and tannin content was expressed as mg catechin equivalent per gram of fresh weight (mg catechin/g F. W.).

#### 2.6.7 Quantitative determination of electrolytic leakage

Electrolyte leakage was quantified to assess membrane stability under stress conditions by using Blum and Ebercon (1981) method. Leaf samples (0.5 g) were excised, washed with distilled water to remove surface-adhered electrolytes and placed in test tubes containing 10 mL of deionized water. The tubes were incubated at 25 °C for 24 h and the initial electrical conductivity (EC₁) was measured using a conductivity meter (HI98129, HANNA Instruments, United States). To ensure complete ion leakage, the samples were autoclaved at 121 °C for 15 min and then allowed to cool to room temperature before recording the final electrical conductivity (EC₂). Electrolyte leakage was then determined using the appropriate Equation 8.


(8) $$\text{Electrolyte leakage}\;(\%)=(\frac{{\mathrm{EC}}_{1}}{{\mathrm{EC}}_{2}})*100$$

#### 2.6.8 Determination of plant enzymes related to plant defense activity

##### 2.6.8.1 Peroxidase

Peroxidase (POX) was determined following the method described by Hammerschmidt et al. (1982). Fresh leaf tissue (1 g) was homogenized in 1 mL of 0.1 M phosphate buffer (pH 7.0) and centrifuged at 15,000 rpm for 15 min at 4 °C. The resulting supernatant was used as the enzyme extract. The reaction mixture consisted of 1.5 mL of 0.05 M pyrogallol, 0.5 mL of 1% hydrogen peroxide (H₂O₂) and 0.1 mL of the enzyme extract. Absorbance was recorded at 420 nm at 30-s intervals over duration of 3 min. Peroxidase activity was expressed as the rate of absorbance change per minute per gram of tissue or U/min/g F. W.

##### 2.6.8.2 Polyphenol oxidase

Polyphenol oxidase (PPOX) was determined following the method described by Harel et al. (1965). Fresh leaf tissue (1 g) was homogenized in 1 mL of 0.1 M sodium phosphate buffer (pH 6.5) and centrifuged at 15,000 rpm for 15 min at 4 °C. The enzyme extract was obtained from the supernatant. The reaction mixture consisted of 1.5 mL of 0.1 M sodium phosphate buffer, 0.1 mL of enzyme extract and 0.2 mL of 0.01 M catechol. Absorbance was recorded at 495 nm at 30-s intervals over a period of 3 min. Enzyme activity was expressed as the rate of change in absorbance per minute per gram of tissue or U/min/g F. W.

##### 2.6.8.3 Phenylalanine ammonia-lyase

Dogbo et al. (2012) method was followed for determination of phenylalanine ammonia-lyase (PAL) activity. Fresh leaf tissue (500 mg) was homogenized in 5 mL of ice-cold 25 mM borate-HCl buffer (pH 8.8) containing 5 Mm mercaptoethanol. The reaction mixture comprised 0.2 mL of enzyme extract, 1.3 mL of distilled water, 0.5 mL of borate buffer and 1 mL of 1 mM L-phenylalanine. After incubation at 32 °C for 1 h, the reaction was halted by adding 0.5 mL of 2 N HCl. Absorbance was measured at 290 nm and enzyme activity was expressed as the amount of cinnamic acid produced per hour per gram of tissue or (U/h/g F. W.).

##### 2.6.8.4 Tyrosine ammonia lyase

Tyrosine ammonia lyase (TAL) activity was measured following a modified method of Mahatma et al. (2019). Fresh leaf tissue (500 mg) was homogenized in 5 mL of ice-cold 25 mM borate-HCl buffer (pH 8.8) containing 5 Mm mercaptoethanol. The reaction mixture comprised 0.2 mL of enzyme extract, 1.3 mL of distilled water, 0.5 mL of borate buffer and 1 mL of 1 mM L-tyrosine. The mixture was incubated at 37 °C for 1 h and the reaction was terminated by adding 0.5 mL of 2 N HCl. Absorbance was measured at 310 nm to quantify the formation of p-coumaric acid. TAL activity was expressed as U/h/g fresh weight (F. W.), where one unit (U) of enzyme activity corresponds to the amount of enzyme required to produce 1 μmol of p-coumaric acid per hour per gram of fresh tissue.

##### 2.6.8.5 Catalase

Catalase (CAT) activity was measured following the method described by Aebi (1984). Fresh leaf tissue (500 mg) was homogenized in 5 mL of ice-cold 50 mM phosphate buffer (pH 7.0) and centrifuged at 15,000 rpm for 15 min at 4 °C. The supernatant was used as the enzyme extract. The reaction mixture comprised 2 mL of 50 mM phosphate buffer (pH 7.0), 0.5 mL of 100 mM H₂O₂ and 0.1 mL of enzyme extract. The decrease in absorbance due to H₂O₂ degradation was recorded at 240 nm at 30-s intervals for 3 min. Catalase activity was expressed as μmol of H₂O₂ decomposed per minute per gram of fresh weight (U/min/g F. W.).

##### 2.6.8.6 Superoxide dismutase

Superoxide dismutase (SOD) activity was determined based on the inhibition of nitro blue tetrazolium (NBT) reduction as described by Beauchamp and Fridovich (1971). Fresh leaf tissue (500 mg) was homogenized in 5 mL of ice-cold 50 mM phosphate buffer (pH 7.8) containing 1% polyvinylpyrrolidone (PVP) and centrifuged at 15,000 rpm for 15 min at 4 °C. The supernatant served as the enzyme extract. The reaction mixture (3 mL) comprised 2.00 mL of 50 mM phosphate buffer (pH 7.8), 0.3 mL of 13 mM methionine, 0.3 mL of 75 μM NBT, 0.3 mL of 2 μM riboflavin and 0.1 mL enzyme extract. The tubes were exposed to fluorescent light for 15 min to induce the photoreduction of NBT. Absorbance was measured at 560 nm and one unit of SOD activity was defined as the amount of enzyme required to inhibit 50% of NBT reduction. Results were expressed as U/g F. W.

#### 2.6.9 Histo-chemical detection of peroxide and superoxide radicals

The accumulation of hydrogen peroxide (H₂O₂) and superoxide (O₂^−^) radicals in plant tissues was detected using 3,3′-diaminobenzidine (DAB) and nitro blue tetrazolium (NBT) staining, respectively (Fryer et al., 2002). For the detection of H₂O₂, fresh leaf samples were immersed in a 1 mg/mL DAB solution prepared in 10 mM phosphate buffer (pH 3.8). The samples were incubated in the dark at 25 °C for 4–6 h, followed by rinsing with distilled water. To remove chlorophyll, the stained samples were boiled in 95% ethanol for 10 min. The presence of H₂O₂ was indicated by the formation of brown precipitates. For the detection of superoxide radicals (O₂^−^), fresh leaf samples were immersed in a 0.1% (w/v) NBT solution prepared in 10 mM phosphate buffer (pH 7.5) and incubated in the dark at 25 °C for 2–4 h. After incubation, the samples were washed with water and decolorized by boiling in 95% ethanol for 10 min to remove chlorophyll. The formation of blue formazan deposits indicated the presence of superoxide radicals.

#### 2.6.10 Plant growth, biomass and yield

Plant height and biomass were evaluated at 70 days after sowing (DAS) to determine the impact of treatments on crop performance. Plant height was recorded using a measuring scale. Fresh weight was measured immediately, while dry weight was recorded after oven-drying the samples at 70 °C until a constant weight was achieved. Yield components were determined at the time of harvest. The numbers of pods per plant were recorded. The total grain yield per plant and per hectare was calculated based on the final seed weight.

### 2.7 Validation of microbial consortia under field experiment

A field experiment was conducted during the Kharif 2024 season at ICAR-Central Arid Zone Research Institute, Jodhpur, to assess the effectiveness of promising microbial consortia in managing *Macrophomina* root rot in cluster bean and its impact on plant growth, biomass and yield (Supplementary Figure S2). The experiment followed a randomized block design (RBD) with five replications and included four treatments: T1 (Uninoculated Control or mock control), T2 (Macrophomina-Infected Control), T3 (*Pseudomonas* sp. 8B + *A. aneurinilyticus* 16B + *B. velezensis* 32B + challenged with *M. phaseolina*), and T4 (*Pseudomonas* sp. 8B + *A. aneurinilyticus* 16B + *B. velezensis* 32B + *T. breve* 37F + challenged with *M. phaseolina*).

Cluster bean (Pusa Navbahar) seeds were sown on 14 July 2024 and treated with biocontrol agents at 6 g/kg (talc-based formulation), while control seeds received blank talc powder. Before treatment, seeds were surface-sterilized (Minchev et al., 2021) and air-dried for 2 h. The sowing was done manually at a 45 cm row spacing, with a seed rate of 15 kg/ha. Fertilization included 20 kg N and 40 kg P₂O₅ per hectare (Singh, 2014), applied as a basal dose using urea and DAP. Additionally, biocontrol agents were applied to the soil at 10 kg/ha at 20 and 30 DAS.

*M. phaseolina* was introduced in T2–T4 treatments on the 40th day using sorghum-based inoculum at 1 kg/plot. Standard agronomic practices were followed, except for the targeted disease management interventions. Plant growth parameters, including height, fresh weight and dry weight, were recorded 70 days after sowing, using five randomly selected plants per plot. Yield was also measured. The crop was harvested on 10th November, 2024.

### 2.8 Statistical analysis

The experimental data were analyzed by calculating mean values and standard deviations (SD) based on the respective replicates. Statistical analysis was performed using the Fisher’s Least Significant Difference (LSD) method at a 95% confidence level (*p* ≤ 0.05) to compare mean values and identify significant differences between treatments. To visualize the antagonistic activity of bacterial and fungal isolates against *M. phaseolina*, a heat map was generated using R software (R-4.4.3) and the “pheatmap” package. The heat map was constructed using hierarchical clustering with Euclidean distance and Ward’s linkage method. The color gradient of the heat map was set to represent inhibition levels, where higher inhibition was indicated by red and lower inhibition by blue.

## 3 Results

### 3.1 Screening of microbial isolates for antagonistic activity against *Macrophomina phaseolina*

Among 283 fungal and 480 bacterial isolates screened, 36 fungal and 73 bacterial isolates showed antagonistic activity against *M. phaseolina* in dual plate assays. Seventeen fungal isolates, including 1F, 2F, 3F, 4F, 6F–15F, 37F, 43F and 44F, recorded the highest inhibition (87.5%), followed by 42F (81.25%), 19F and 41F (75%), while isolates 5F and 21F showed moderate inhibition (62.5%). Lower inhibition was noted in 20F (50%) and 40F (47.5%), with the weakest activity in 22F (7.5%) and 23F (8.75%) (Supplementary Table S1). Among bacteria, maximum inhibition (87.5%) was observed in 12 isolates (8B, 10B, 16B, 20B, 32B, 34B, 48B, 72B, 169B, 248B, 301B, 302B), followed by 271B (78.75%), 17B and 197B (77.5%), and nine isolates with 75% inhibition. Moderate activity (67.5–70%) was recorded in nine isolates, while several others showed lower inhibition (50–65%). The least inhibition was observed in 267B (40%) and 477B (37.5%) (Supplementary Table S2).

### 3.2 Heat map analysis of functional traits

**Figure 1:**
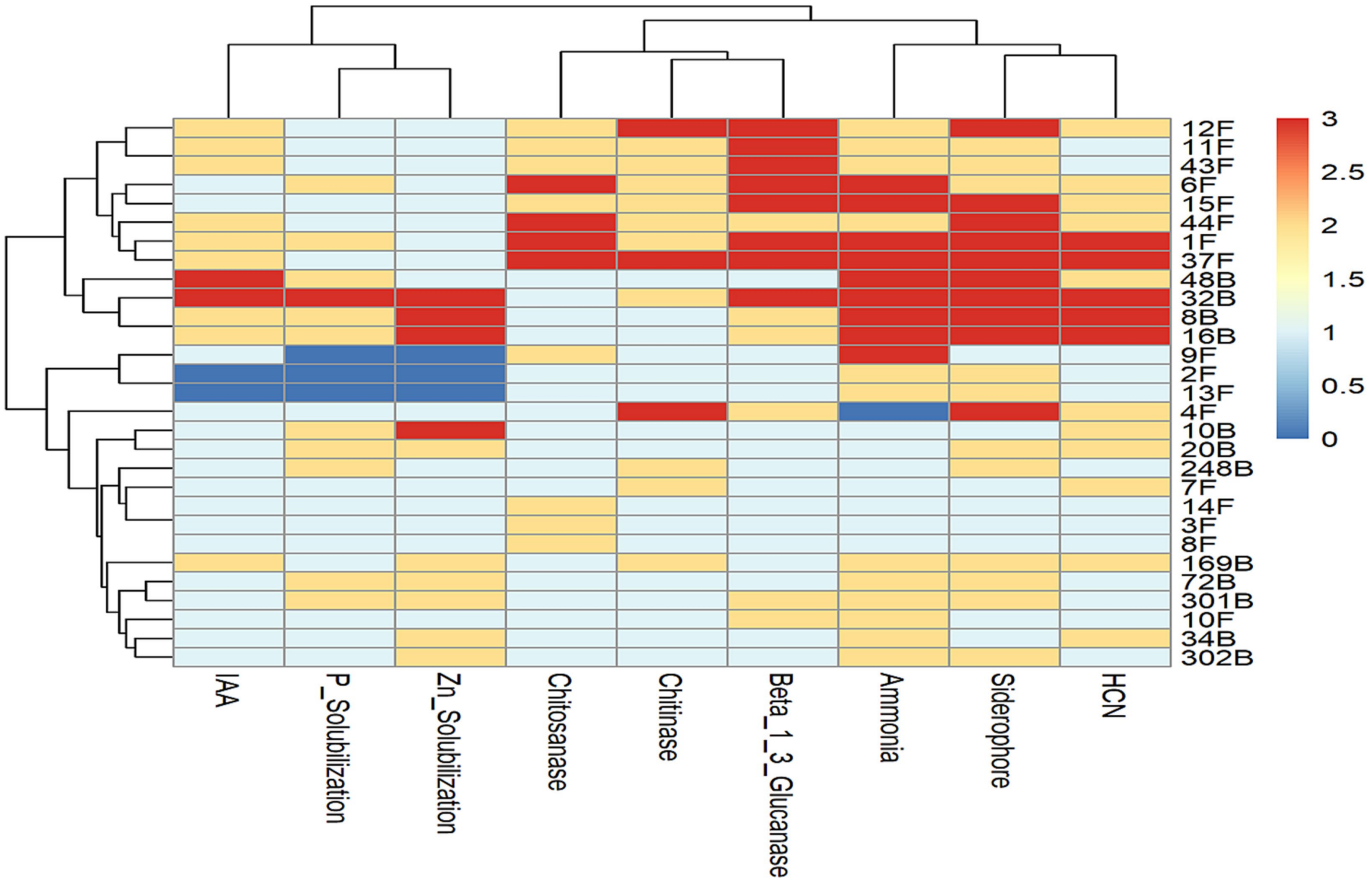
Heat map illustrating biocontrol and plant growth promoting activities of selected promising biocontrol agents.

The heat map revealed clear clustering of fungal and bacterial isolates based on antagonistic and plant growth-promoting traits (Figure 1). Potent isolates such as 37F, 32B and 16B clustered together due to high production of siderophore, HCN, ammonia and chitinase, whereas low-performing isolates (e.g., 2F, 3F, 9F) formed a separate group. Trait co-occurrence was evident, with siderophore, HCN, and ammonia clustering together, while chitinase, β-1,3-glucanase and chitosanase formed another cluster. Isolates 37F, 32B, 16B and 8B expressed multiple PGPR traits and exhibited strong phosphate and zinc solubilization, making them the top candidates for further evaluation.

### 3.3 *In vitro* compatibility of selected biocontrol agents

**Figure 2:**
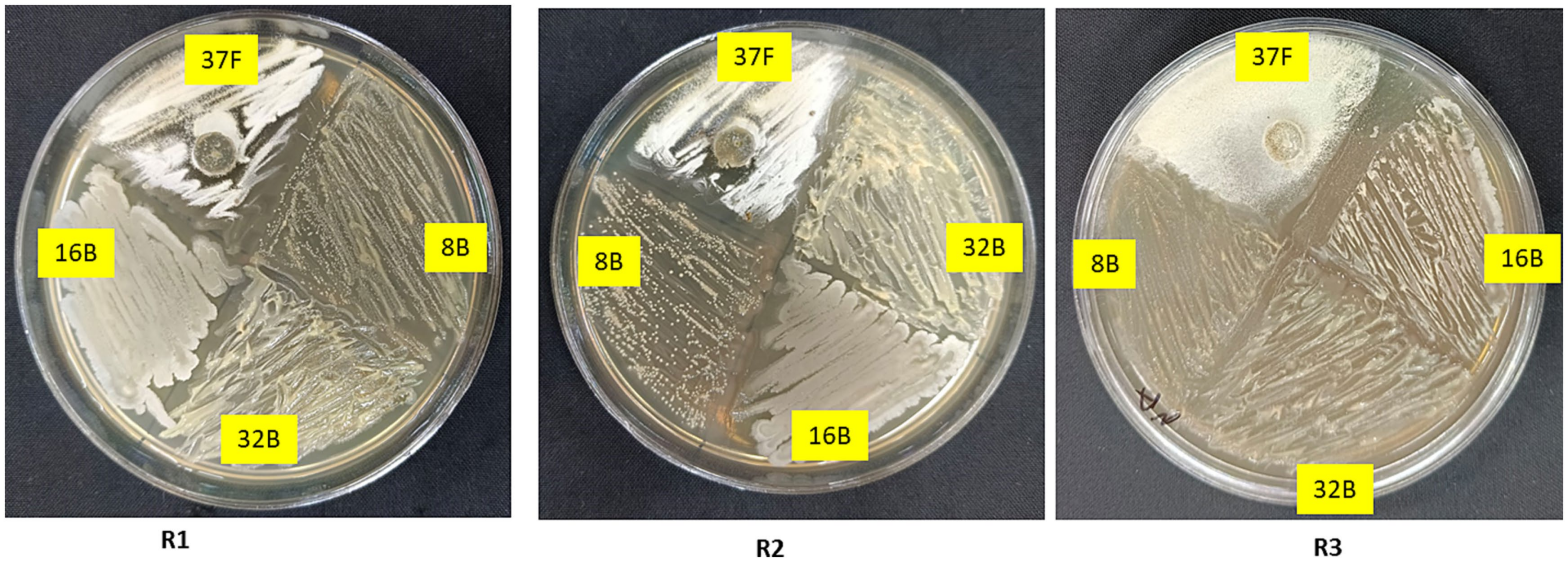
*In vitro* compatibility test of selected biocontrol agents with each other.

The compatibility of *T. breve* 37F with bacterial isolates 8B, 16B and 32B was successfully evaluated using the cross-streak method (Figure 2). After 5 days of incubation, all isolates exhibited uninterrupted growth in close proximity without the formation of inhibition zones or adverse interactions. The bacterial isolates did not show any suppression of 37F and vice versa, indicating a high level of mutual compatibility. Additionally, there were no observable changes in colony morphology, further confirming their ability to coexist. Therefore, *T. breve* 37F and the bacterial isolates 8B, 16B and 32B can be effectively combined for potential biocontrol applications, as their interaction did not lead to antagonism.

### 3.4 Characterization of selected biocontrol agents: morphological, physiological and biochemical aspects

Morphological analysis of the bacterial isolates (8B, 16B, 32B) showed medium-sized, circular colonies with distinct variations in margin, opacity, and pigmentation (Supplementary Table S3). Isolates 8B and 16B had entire margins, while 32B showed slightly undulate margins. Pigmentation differed, with 8B greenish-yellow and 16B/32B white. Gram staining classified 8B as Gram-negative and 16B/32B as Gram-positive; all were rod-shaped, with endospore formation in 16B and 32B only. *T. breve* 37F exhibited typical *Trichoderma* features, including septate hyphae, branched conidiophores with flask-shaped phialides, ellipsoidal to globose conidia, and rapid colony growth that transitioned from white to green/yellow (Supplementary Table S4).

Physiological and biochemical profiling revealed all bacterial isolates metabolized several sugars (e.g., xylose, maltose, fructose, galactose, trehalose, sucrose) but not raffinose, inulin, or dulcitol. Isolate 8B uniquely utilized lactose and melibiose, whereas 16B and 32B utilized salicin, sorbitol, and cellobiose. Esculin hydrolysis, lysine/ornithine utilization occurred in 16B and 32B but not 8B. Urease was detected only in 8B. All isolates tested positive for citrate, nitrate reductase, oxidase, casein hydrolysis and catalase, while negative for malonate, ONPG, phenylalanine deamination, and H₂S production. KOH confirmed 8B as positive and 16B/32B as negative (Supplementary Table S5).

### 3.5 Molecular identification and phylogenetic analysis of selected isolates

**Figure 3:**
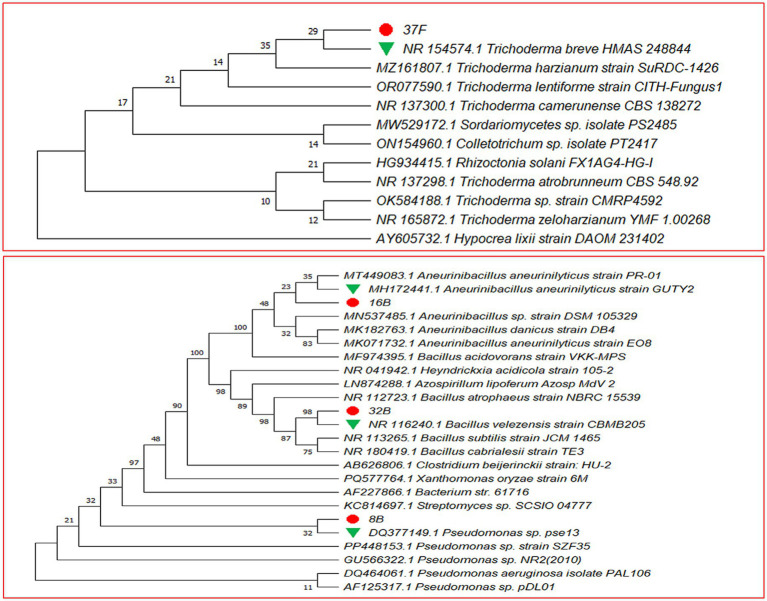
Phylogenetic tree constructed using Maximum Likelihood method based on Tamura-Nei model (1993) with 1,000 Bootstrap replications for promising biocontrol agents derived from Clustal W alignment of 16S rDNA partial sequences.

The phylogenetic analysis based on ITS and 16S rRNA gene sequences confirmed the taxonomic identity of the selected isolates (Figure 3). Isolate 37F clustered closely with *T. breve* HMAS (Accession No. 248844), indicating a strong genetic affiliation with this species. Similarly, bacterial isolates 8B, 16B and 32B formed distinct clades with their respective closest relatives. Isolate 8B grouped closely with Pseudomonas sp. pse13 (Accession No. DQ377149), while 16B clustered with *A. aneurinilyticus* strain GUTY2 (Accession No. MH172441). Isolate 32B formed a monophyletic cluster with *B. velezensis* strain CBMB 205 (Accession No. NR116240). The ITS-based sequence analysis of isolate 37F revealed 100% similarity with T. breve HMAS. Isolate 8B exhibited 99.40% similarity with Pseudomonas sp. pse13, while 16B showed 98.14% similarity with A.aneurinilyticus strain GUTY2. Isolate 32B displayed 99.48% similarity with *B. velezensis* strain CBMB 205. The identified sequences were submitted to the NCBI GenBank database and assigned accession numbers OR105515 (37F), PP869086 (8B), PP064118 (16B) and PP863874 (32B).

### 3.6 Assessment of biocontrol efficacy of selected agents, individually and in consortium, against *Macrophomina phaseolina*

The bioefficacy evaluation of biocontrol agents against *M. phaseolina* causes dry root rot in cluster bean revealed significant differences among treatments (*p* ≤ 0.05). The highest disease suppression (87.13%) was observed in the consortium treatment of *T. breve* 37F + *Pseudomonas* sp. 8B + *A. aneurinilyticus* 16B + *B. velezensis* 32B (T17), which significantly reduced the PDI to 16.7%. Among dual and triple combinations, 37F + 16B + 32B (T15) exhibited 77.3% disease control, followed by 37F + 8B + 16B and 37F + 8B + 32B with 72.7% control (Table 1). Individual treatments showed moderate disease reduction, with 37F alone (T3) providing 54.5% control, significantly better than 8B (T4) and 16B (T5) (both 31.8%). The infected control (T2) had the highest PDI (91.7%), confirming the pathogenicity of *M. phaseolina*. Overall, consortium treatments were significantly more effective than individual strains in controlling disease severity.

### 3.7 Impact of biocontrol agents, individually and in consortium, on chlorophyll and carotenoid content in cluster bean against *Macrophomina phaseolina*

The effect of biocontrol agents on chlorophyll and carotenoid content in cluster bean showed significant differences among treatments (*p* ≤ 0.05). The highest chlorophyll a, chlorophyll b and total chlorophyll were recorded in the four microbe consortium treatment of *T. breve* 37F + *Pseudomonas* sp. 8B + *A.aneurinilyticus* 16B + *B. velezensis* 32B (T17), which was significantly superior to all other treatments (Table 2). Among individual treatments, 16B (T5) and 32B (T6) showed relatively higher chlorophyll content, but they were significantly lower than consortium treatments. Carotenoid content remained statistically similar across most treatments, with T17 recording the highest value. When compared to the infected control, T17 demonstrated a 2.72-fold increase in chlorophyll a, a 3.59-fold increase in chlorophyll b, a 3.16-fold increase in total chlorophyll and a 1.95-fold increase in carotenoid content. Similarly, in comparison to the mock control (T1), T17 exhibited a 1.53-fold increase in chlorophyll a, a 2.20-fold increase in chlorophyll b, a 2.18-fold increase in total chlorophyll, and a 1.10-fold increase in carotenoids.

**Table 2:** Effect of biocontrol agents in individual and consortium mode on chlorophyll and carotenoids content of cluster bean against *Macrophomina phaseolina* of cluster bean in pot experiment during 2023.

Treatments	Chlorophyll a (mg/g F. W.)	Chlorophyll b (mg/gF. W.)	Total chlorophyll (mg/g F. W.)	Carotenoids (mg/g F. W.)
T1: No pathogen + No biocontrol agent (mock control)	0.96 ± 0.06^f^	0.44 ± 0.10^def^	1.29 ± 0.10^h^	0.39 ± 0.10^a^
T2: Only *Macrophomina phaseolina* (infected control)	0.54 ± 0.10^g^	0.27 ± 0.03^f^	0.89 ± 0.10^i^	0.22 ± 0.04^b^
T3: *Trichoderma breve* 37F + challenged with *M. phaseolina*	0.98 ± 0.02^ef^	0.44 ± 0.07^def^	1.23 ± 0.13^h^	0.33 ± 0.05^ab^
T4: *Pseudomonas* sp. 8B + challenged with *M. phaseolina*	1.13 ± 0.07^def^	0.53 ± 0.15^cde^	1.61 ± 0.20^g^	0.30 ± 0.03^ab^
T5: *Aneurinibacillus aneurinilyticus* 16B + challenged with *M. phaseolina*	1.27 ± 0.27^abcd^	0.38 ± 0.08^ef^	1.72 ± 0.20^fg^	0.34 ± 0.04^ab^
T6: *Bacillus velezensis* 32B + challenged with *M. phaseolina*	1.24 ± 0.13^abcd^	0.33 ± 0.08^f^	1.67 ± 0.20^fg^	0.33 ± 0.06^ab^
T7: 37F + 8B + challenged with *M. phaseolina*	1.27 ± 0.12^abcd^	0.61 ± 0.21^cd^	1.94 ± 0.10^def^	0.38 ± 0.13^a^
T8: 37F + 16B + challenged with *M. phaseolina*	1.29 ± 0.09^abcd^	0.70 ± 0.10^c^	2.05 ± 0.15^cde^	0.39 ± 0.09^a^
T9: 37F + 32B + challenged with *M. phaseolina*	1.25 ± 0.15^abcd^	0.57 ± 0.10^cd^	1.86 ± 0.10^de^fg	0.42 ± 0.10^a^
T10: 8B + 16B + challenged with *M. phaseolina*	1.17 ± 0.17^cdef^	0.68 ± 0.10^c^	1.94 ± 0.10^def^	0.38 ± 0.04^a^
T11: 8B + 32B + challenged with *M. phaseolina*	1.20 ± 0.10^bcdef^	0.60 ± 0.10^cd^	1.79 ± 0.10^efg^	0.38 ± 0.10^a^
T12: 16B + 32B + challenged with *M. phaseolina*	1.22 ± 0.12^bcde^	0.61 ± 0.11^cd^	1.90 ± 0.20^def^	0.40 ± 0.09^a^
T13: 37F + 8B + 16B + challenged with *M. phaseolina*	1.33 ± 0.18^abcd^	0.71 ± 0.10^bc^	2.12 ± 0.12^cd^	0.41 ± 0.04^a^
T14: 37F + 8B + 32B + challenged with *M. phaseolina*	1.34 ± 0.17^abcd^	0.68 ± 0.10^c^	2.05 ± 0.10^cde^	0.43 ± 0.13^a^
T15: 37F + 16B + 32B + challenged with *M. phaseolina*	1.42 ± 0.22^ab^	0.68 ± 0.10^c^	2.26 ± 0.16^bc^	0.41 ± 0.10^a^
T16: 8B + 16B + 32B + challenged with *M. phaseolina*	1.40 ± 0.10^abc^	0.88 ± 0.10^ab^	2.40 ± 0.30^b^	0.40 ± 0.10^a^
T17: 37F + 8B + 16B + 32B + challenged with *M. phaseolina*	1.47 ± 0.20^a^	0.97 ± 0.10^a^	2.81 ± 0.30^a^	0.43 ± 0.07^a^

*Data are the average of three replicates ± SD; Grouping information between mean values of obtained data was carried out by Fisher LSD Method and 95% confidence (*P* ≤ 0.05). Different letter point out significant differences in a column.

### 3.8 Influence of biocontrol agents, individually and in consortium, on biochemical traits of cluster bean under *Macrophomina phaseolina* stress

The application of biocontrol agents, both in individual and consortium modes, significantly influenced the biochemical attributes of cluster bean under *M. phaseolina* infection in the pot experiment over the infected control. The results demonstrated a progressive enhancement in biochemical attributes with increasing microbial diversity in consortia treatments. The highest total phenol accumulation was recorded in four microbe consortium treatment, which was statistically at par with three bacterial consortium treatments. The four-microbe consortium treatment enhanced total phenol content by 2.1-fold over the infected control (Table 3). Among the three-microbe consortia, T16 (7.70 mg gallic acid/g F. W.) and T15 (6.9 mg gallic acid/g F. W.) recorded significantly higher phenol content than two-microbe consortia. The highest phenol content among individual treatments was recorded with *T. breve* 37F which was 1.4-fold higher than the infected control.

**Table 3:** Effect of biocontrol agents in individual and consortium mode on biochemical characteristics of cluster bean against *Macrophomina phaseolina* of cluster bean in pot experiment during 2023.

Treatments	Total phenol (mg gallic acid/g F. W.)	Flavonoids (mg quercitin/g F. W.)	Antioxidant (mM Trolox/g F. W.)	Tannin (mg catechin/g F. W.)	Electrolytic leakage (%)
T1: No pathogen + No biocontrol agent (mock control)	2.0 ± 0.38^f^	16.2 ± 1.31^d^	2.43 ± 0.36^ef^	1.23 ± 0.32^h^	63.83 ± 12^ab^
T2: Only *Macrophomina phaseolina* (infected control)	3.7 ± 0.87^ef^	22.3 ± 3.5^d^	2.07 ± 0.87^f^	2.23 ± 0.41^gh^	85.36 ± 21^a^
T3: *Trichoderma breve* 37F + challenged with *M. phaseolina*	5.3 ± 0.56^bcde^	48.8 ± 2.74^b^	2.88 ± 0.75^bcdef^	3.43 ± 0.62^fg^	66.86 ± 15^ab^
T4: *Pseudomonas* sp. 8B + challenged with *M. phaseolina*	4.4 ± 0.63^cde^	40.2 ± 2.68^c^	2.89 ± 0.40^bcdef^	3.40 ± 0.75^fg^	63.42 ± 12^b^
T5: *Aneurinibacillus aneurinilyticus* 16B + challenged with *M. phaseolina*	4.2 ± 0.81^de^	40.2 ± 4.20^c^	2.70 ± 0.39^cdef^	3.63 ± 0.65^efg^	66.77 ± 11^ab^
T6: *Bacillus velezensis* 32B + challenged with *M. phaseolina*	4.5 ± 1.9^cde^	40.5 ± 4.41^c^	2.65 ± 0.52^def^	3.96 ± 0.45^def^	61.27 ± 10^b^
T7: 37F + 8B + challenged with *M. phaseolina*	6.1 ± 1.2^abcd^	54.8 ± 4.40^ab^	3.34 ± 0.75^bcde^	5.73 ± 0.29^c^	58.94 ± 09^b^
T8: 37F + 16B + challenged with *M. phaseolina*	6.4 ± 1.1^abc^	56.7 ± 5.40^a^	3.37 ± 0.72^bcde^	5.63 ± 0.87^c^	55.60 ± 08^b^
T9: 37F + 32B + challenged with *M. phaseolina*	6.0 ± 1.17^abcd^	57.1 ± 4.20^a^	3.30 ± 0.36^bcde^	5.36 ± 0.87^cd^	52.68 ± 19^b^
T10: 8B + 16B + challenged with *M. phaseolina*	6.3 ± 1.10^abc^	54.2 ± 4.21^ab^	3.14 ± 0.86^bcdef^	5.03 ± 1.23^cde^	50.18 ± 17^b^
T11: 8B + 32B + challenged with *M. phaseolina*	6.1 ± 1.1^abcd^	52.2 ± 4.35^ab^	3.11 ± 0.97^bcdef^	5.19 ± 1.25^cd^	59.47 ± 18^b^
T12: 16B + 32B + challenged with *M. phaseolina*	6.1 ± 1.3^abcd^	51.9 ± 5.45^ab^	3.08 ± 1.0^bcdef^	5.36 ± 1.36^cd^	56.71 ± 08^b^
T13: 37F + 8B + 16B + challenged with *M. phaseolina*	7.3 ± 1.9^ab^	56.7 ± 6.51^a^	3.69 ± 0.56^bcd^	7.83 ± 1.5^b^	56.08 ± 09^b^
T14: 37F + 8B + 32B + challenged with *M. phaseolina*	7.1 ± 1.2^ab^	56.4 ± 7.23^a^	3.79 ± 0.56^bc^	7.83 ± 1.2^b^	50.23 ± 11^b^
T15: 37F + 16B + 32B + challenged with *M. phaseolina*	6.9 ± 1.1^ab^	54.5 ± 3.38^ab^	3.80 ± 0.45^bc^	8.06 ± 0.89^b^	46.56 ± 10^b^
T16: 8B + 16B + 32B + challenged with *M. phaseolina*	7.7 ± 1.5^a^	57.4 ± 4.31^a^	4.27 ± 1.0^b^	8.89 ± 0.56^b^	45.76 ± 12^b^
T17: 37F + 8B + 16B + 32B + challenged with *M. phaseolina*	7.8 ± 1.6^a^	56.3 ± 5.54^ab^	6.65 ± 0.9^a^	10.53 ± 0.84^a^	45.42 ± 07^b^

*Data are the average of three replicates ± SD; Grouping information between mean values of obtained data was carried out by Fisher LSD Method and 95% confidence (*P* ≤ 0.05). Different letter point out significant differences in a column.

Flavonoid accumulation also showed a significant increase with microbial consortia. The maximum flavonoid content was recorded in three bacterial consortium comprising *Pseudomonas* sp. 8B + *A. aneurinilyticus* 16B + *B. velezensis* 32B treatment followed by *T. breve* 37F + *B. velezensis* 32B consortium, which was 2.6-fold higher than the infected control. Among individual applications, *T. breve* 37F recorded the highest flavonoid which was 2.2-fold higher than the infected control (Table 3). The highest antioxidant activity was observed in four-microbe consortium, which was 3.2-fold higher than the infected control. Three-microbe consortia recorded antioxidant activity in the range of 3.69–4.27 mM Trolox/g F. W., while two-microbe combinations ranged between 3.08–3.37 Mm Trolox/g F. W. Among individual applications, *T. breve* 37F had the highest antioxidant activity which was 1.4-fold higher than the infected control but significantly lower than consortia treatments (Table 3).

Tannin accumulation was significantly enhanced in consortia treatments, with the highest content recorded in the four-microbe consortium, which was 4.7-fold higher than the infected control. Three-microbe consortia exhibited 3.5–3.9-fold higher tannin content than the infected control. Two-microbe consortia generally showed 2.3–2.5-fold higher values than the infected control. The highest tannin content among individual treatments was observed in *B. velezensis* 32B (3.96 mg catechin/g F. W.), 1.8-fold higher than the infected control but significantly lower than two-, three- and four-microbe consortia (Table 3).

Electrolyte leakage was significantly reduced in biocontrol-treated plants. The highest reduction was observed in the four-microbe consortium (45.42%) followed by T16 treatment (45.76%). T17 treatment showed a 46.8% decrease compared to the infected control. Three-microbe consortia exhibited 45.5–46.4% lower electrolyte leakage than the infected control, whereas two-microbe consortia showed a 38.3–41.2% reduction. Among individual applications, *B. velezensis* 32B recorded the lowest electrolyte leakage, which was 28.3% lower than the infected control but significantly higher than consortia treatments (Table 3).

### 3.9 Influence of biocontrol agents, individually and in consortium, on antioxidant defense enzymes of cluster bean under *Macrophomina phaseolina* stress

The antioxidant defense enzyme activities in cluster bean were significantly influenced by different biocontrol treatments in both individual and consortium modes when challenged with *M. phaseolina*. The combined application of biocontrol agents resulted in a greater enhancement of enzyme activity compared to individual treatments. For POX, the highest enzyme induction was observed in the four-microbe consortium treatment with 8.5 U/min/g F. W., which was statistically at par with the T9 (*T. breve* 37F + *B. velezensis* 32B) that recorded 8.2 U/min/g F. W. These two treatments were superior to all other two-microbe and three-microbe consortia. T17 treatment exhibited 4.05-fold higher POX over the infected control (Table 4). PPOX activity was recorded highest in T16 treatment being statistically similar with T17. T16 treatment exhibited 2.90-fold higher PPOX activity over the infected control (Table 4).

**Table 4:** Effect of biocontrol agents in individual and consortium mode on antioxidant defense enzymes of clusterbean against *Macrophomina phaseolina* of cluster bean in pot experiment during 2023.

Treatments	Peroxidase (U/min./g F. W.)	Polyphenol oxidase (U/min./g F. W.)	Phenylalanine ammonia lyase (U/h/g F. W.)	Tyrosine ammonia lyase (U/h/g F. W.)	Superoxide dismutase (U/g F. W.)	Catalase (U/min/g F. W.)
T1: No pathogen + No biocontrol agent (mock control)	1.3 ± 0.3^f^	9.9 ± 1.1^d^	3.1 ± 0.6^b^	23.47 ± 1.4^f^	0.12 ± 0.05^f^	2.44 ± 1.0^c^
T2: Only *Macrophomina phaseolina* (infected control)	2.1 ± 0.4^f^	13.2 ± 1.0^d^	4.5 ± 0.2^b^	29.4 ± 2.9^ef^	0.17 ± 0.06^ef^	3.06 ± 0.20^c^
T3: *Trichoderma breve* 37F + challenged with *M. phaseolina*	5.7 ± 0.7^cde^	20.1 ± 1.2^c^	8.6 ± 0.8^a^	54.5 ± 8.0^a^	0.32 ± 0.08^cd^	5.51 ± 0.99^ab^
T4: *Pseudomonas* sp. 8B + challenged with *M. phaseolina*	4.9 ± 0.5^de^	20.2 ± 2.07^c^	7.5 ± 0.9^a^	32.2 ± 7.0^cde^	0.36 ± 0.09^bcd^	4.81 ± 0.78^b^
T5: *Aneurinibacillus aneurinilyticus* 16B + challenged with *M. phaseolina*	4.8 ± 0.8^e^	21.4 ± 2.8^c^	7.5 ± 0.4^a^	31.4 ± 5.4^def^	0.36 ± 0.07^bcd^	5.06 ± 0.93^b^
T6: *Bacillus velezensis* 32B + challenged with *M. phaseolina*	6.4 ± 0.8^bcd^	23.3 ± 2.4^c^	7.6 ± 0.9^a^	35.1 ± 5.1^bcde^	0.30 ± 0.08^de^	5.04 ± 1.01^b^
T7: 37F + 8B + challenged with *M. phaseolina*	4.7 ± 1.0^e^	33.0 ± 3.5^ab^	8.5 ± 1.0^a^	41.3 ± 4.1^b^	0.39 ± 0.09^abcd^	5.75 ± 1.09^ab^
T8: 37F + 16B + challenged with *M. phaseolina*	5.6 ± 0.5^cde^	32.2 ± 4.4^ab^	8.6 ± 1.5^a^	41.1 ± 3.1^b^	0.43 ± 0.10^abcd^	5.85 ± 1.03^ab^
T9: 37F + 32B + challenged with *M. phaseolina*	8.2 ± 1.6^a^	33.0 ± 3.3^ab^	8.5 ± 1.0^a^	42.2 ± 3.4^b^	0.45 ± 0.10^abc^	5.91 ± 0.86^ab^
T10: 8B + 16B + challenged with *M. phaseolina*	5.8 ± 1.11^cde^	31.3 ± 4.3^ab^	7.3 ± 1.1^a^	37.1 ± 4.1^bcdef^	0.43 ± 0.06^abcd^	5.85 ± 0.90^ab^
T11: 8B + 32B + challenged with *M. phaseolina*	5.9 ± 0.8^cde^	34.3 ± 5.6^ab^	7.8 ± 1.6^a^	38.4 ± 4.1^bcd^	0.42 ± 0.04^abcd^	5.64 ± 0.87^ab^
T12: 16B + 32B + challenged with *M. phaseolina*	5.8 ± 1.10^cde^	35.2 ± 4.7^ab^	7.5 ± 1.5^a^	39.4 ± 2.1^bc^	0.40 ± 0.10^abcd^	5.76 ± 1.00^ab^
T13: 37F + 8B + 16B + challenged with *M. phaseolina*	5.9 ± 1.10^cde^	36.3 ± 3.0^ab^	7.6 ± 1.5^a^	59.1 ± 3.1^a^	0.46 ± 0.08^ab^	5.96 ± 1.21^ab^
T14: 37F + 8B + 32B + challenged with *M. phaseolina*	5.9 ± 1.0^cde^	31.2 ± 6.4^b^	8.5 ± 1.1^a^	56.4 ± 4.1^a^	0.48 ± 0.12^ab^	6.04 ± 1.30^ab^
T15: 37F + 16B + 32B + challenged with *M. phaseolina*	7.9 ± 1.1^ab^	33.4 ± 4.9^ab^	8.6 ± 1.1^a^	55.3 ± 2.2^a^	0.47 ± 0.10^ab^	6.21 ± 1.19^ab^
T16: 8B + 16B + 32B + challenged with *M. phaseolina*	6.5 ± 1.08^bc^	37.7 ± 4.3^a^	6.8 ± 1.3^a^	38.0 ± 5.1^bcd^	0.49 ± 0.07^ab^	6.81 ± 1.04^a^
T17: 37F + 8B + 16B + 32B + challenged with *M. phaseolina*	8.5 ± 1.0^a^	35.5 ± 5.1^ab^	8.7 ± 1.5^a^	59.0 ± 9.1^a^	0.50 ± 0.09^a^	6.90 ± 1.20^a^

*Data are the average of three replicates ± SD; Grouping information between mean values of obtained data was carried out by Fisher LSD Method and 95% confidence (*P* ≤ 0.05). Different letter point out significant differences in a column.

For PAL activity, all treatments were statistically equal and showed significantly higher values than infected control. The highest PAL activity was recorded in T17 (8.7 U/h/g F. W.), followed by T8 (*T. breve* 37F + *A. aneurinilyticus* 16B) (8.6 U/h/g F. W.). T17 treatment exhibited 1.93 fold higher PAL activities over the infected control (Table 4). For TAL activity, the highest value was recorded in T13 (*T. breve* 37F + *Pseudomonas* sp. 8B + *A. aneurinilyticus* 16B) (59.1 U/h/g F. W.), followed by T17. T13 treatment exhibited 2.0-fold higher TAL activities over the infected control (Table 4).

SOD activity was highest in T17 (0.50 U/g F. W.), followed by T16 (*Pseudomonas* sp. 8B + *A. aneurinilyticus* 16B + *B. velezensis* 32B) (0.49 U/g F. W.) with no statistical differences. T17 treatment exhibited 2.94-fold fold higher SOD activity over the infected control (Table 4). For CAT, the highest value was observed in T17 (6.90 U/min/g F. W.), which was statistically similar to T16, T15 and T14. T17 treatment exhibited 2.26-fold fold higher CAT activity over the infected control (Table 4).

### 3.10 Histo-chemical detection of peroxide and superoxide radicals

**Figure 4:**
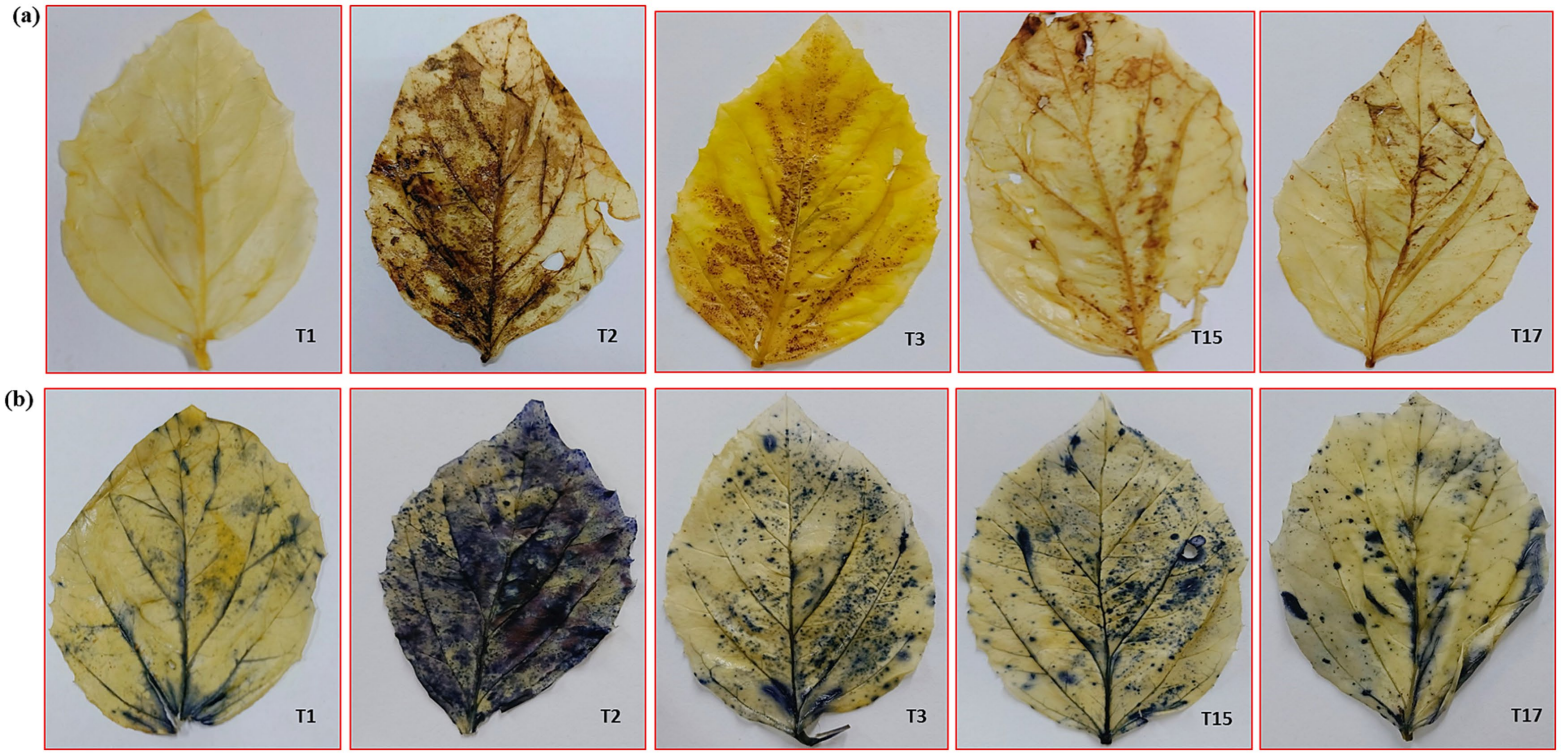
Histo-chemical staining of cluster bean leaves for superoxide **(a)** and peroxide radicals **(b)**.

T2 (Infected control) exhibited the highest intensity of both brown-colored precipitate (peroxide accumulation) and blue staining (superoxide accumulation). In contrast, T1 (Mock control) showed minimal staining. Single biocontrol agent treatments (T3–T6) resulted in a moderate reduction in both brown-colored precipitate (peroxide accumulation) and blue staining (superoxide accumulation) compared to T2. Two-microbe consortia exhibited a further reduction in ROS levels, with T9 (*T. breve* 37F + *B. velezensis* 32B) showing the lowest staining intensity among them. Three-microbe consortia led to a significant reduction of both brown-colored precipitate (peroxide accumulation) and blue staining (superoxide accumulation) compared to two-microbe consortia. The four-microbe consortium (*T. breve* 37F + *Pseudomonas* sp. 8B + *A. aneurinilyticus* 16B + *B. velezensis* 32B) exhibited the least staining intensity for both peroxide and superoxide accumulation (Figures 4A,B).

### 3.11 Impact of biocontrol agents, individually and in consortium, on growth, biomass, yield and yield attributes of cluster bean under *Macrophomina phaseolina* stress

The application of biocontrol agents, both individually and in consortia, significantly improved plant growth, biomass, yield, and yield attributes in cluster bean under *M. phaseolina* infection. Among the individual biocontrol treatments, *T. breve* 37F (T3) exhibited the highest improvement, with plant height increasing by 1.24-fold, fresh weight by 1.70-fold and dry weight by 1.61-fold compared to the infected control (T2). Other single-agent treatments, including *Pseudomonas* sp. 8B (T4), *A. aneurinilyticus* 16B (T5) and *B. velezensis* 32B (T6), also led to improvements, but their effectiveness remained moderate compared to multi-microbe consortia. The increase in the number of pods per plant and yield per pot among these treatments was significant over the infected control; however, individual biocontrol agents alone were not highly effective compared to microbial consortium treatments (Table 5).

**Table 5:** Effect of biocontrol agents in individual and consortium mode on clusterbean growth, biomass, yield and yield attributes against *Macrophomina phaseolina* of cluster bean in pot experiment during 2023.

Treatments	Plant height (cm)	Fresh weight (g)	Dry weight (g)	Number of pods/plant	Yield/pot (g)
T1: No pathogen + No biocontrol agent (mock control)	68.0 ± 2.9^c^	66.5 ± 5.1^bc^	16.3 ± 1.0^d^	19.0 ± 3.4^def^	41.8 ± 5.3^cd^
T2: Only *Macrophomina phaseolina* (infected control)	55.0 ± 6.4^e^	33.5 ± 2.5^g^	9.5 ± 1.3^h^	2.5 ± 5.0^i^	2.7 ± 5.4^g^
T3: *Trichoderma breve* 37F + challenged with *M. phaseolina*	68.2 ± 5.3^c^	56.8 ± 6.9^d^	15.3 ± 1.0^de^	16.5 ± 2.6^fgh^	24.7 ± 4.5^e^
T4: *Pseudomonas* sp. 8B + challenged with *M. phaseolina*	64.8 ± 3.5^cd^	48.3 ± 7.7^ef^	12.5 ± 1.3^fg^	14.5 ± 1.7^h^	14.6 ± 3.9^f^
T5: *Aneurinibacillus aneurinilyticus* 16B + challenged with *M. phaseolina*	68.01.6^c^	47.5 ± 6.5^ef^	12.3 ± 1.7^g^	15.0 ± 2.6^gh^	13.8 ± 2.2^f^
T6: *Bacillus velezensis* 32B + challenged with *M. phaseolina*	60.3 ± 2.8^de^	47.3 ± 5.6^f^	12.3 ± 1.7^g^	14.8 ± 1.7^h^	17.2 ± 8.1^ef^
T7: 37F + 8B + challenged with *M. phaseolina*	68.3 ± 3.9^c^	70.8 ± 7.9^bc^	18.3 ± 1.7^bc^	21.5 ± 1.9^cd^	38.7 ± 8.3^c^
T8: 37F + 16B + challenged with *M. phaseolina*	65.5 ± 4.4^cd^	67.0 ± 5.3^bc^	17.0 ± 1.4^cd^	21.0 ± 2.2^cde^	43.9 ± 8.2^bc^
T9: 37F + 32B + challenged with *M. phaseolina*	68.5 ± 3.4^c^	67.3 ± 5.6^bc^	16.8 ± 1.3^cd^	22.0 ± 0.8^cd^	43.0 ± 4.9^cd^
T10: 8B + 16B + challenged with *M. phaseolina*	66.8 ± 6.3^c^	56.3 ± 8.5^d^	14.3 ± 1.7^ef^	16.5 ± 1.3^fgh^	16.7 ± 4.1^ef^
T11: 8B + 32B + challenged with *M. phaseolina*	63.8 ± 4.5^cd^	57.0 ± 5.7^d^	14.3 ± 1.0^ef^	16.3 ± 1.0^fgh^	18.4 ± 3.9^ef^
T12: 16B + 32B + challenged with *M. phaseolina*	68.8 ± 4.1^c^	55.3 ± 3.6^de^	14.3 ± 1.0^ef^	18.0 ± 0.8^efg^	22.0 ± 6.2^ef^
T13: 37F + 8B + 16B + challenged with *M. phaseolina*	82.3 ± 6.1^b^	70.5 ± 3.1^bc^	18.5 ± 1.3^bc^	22.8 ± 2.1^c^	53.0 ± 9.2^ab^
T14: 37F + 8B + 32B + challenged with *M. phaseolina*	77.5 ± 3.3^b^	74.3 ± 1.7^b^	19.0 ± 0.8^b^	26.0 ± 0.8^b^	54.4 ± 7.2^a^
T15: 37F + 16B + 32B + challenged with *M. phaseolina*	78.0 ± 3.2^b^	71.3 ± 3.6^b^	18.3 ± 1.3^bc^	27.3 ± 1.5^ab^	57.9 ± 11.3^a^
T16: 8B + 16B + 32B + challenged with *M. phaseolina*	80.3 ± 3.0^b^	63.0 ± 6.2^cd^	16.0 ± 1.8^de^	21.5 ± 2.4^cd^	34.4 ± 5.8^d^
T17: 37F + 8B + 16B + 32B + challenged with *M. phaseolina*	91.5 ± 7.9^a^	94.3 ± 3.1^a^	24.3 ± 1.0^a^	29.5 ± 1.0^a^	57.8 ± 7.4^a^

*Data are the average of three replicates ± SD; Grouping information between mean values of obtained data was carried out by Fisher LSD Method and 95% confidence (*P* ≤ 0.05). Different letter point out significant differences in a column.

The two-microbe consortia demonstrated a significant improvement over individual treatments. Among these, *T. breve* 37F + *B. velezensis* 32B (T9) showed the highest enhancement, with plant height increasing by 1.25-fold, fresh weight by 2.01-fold and dry weight by 1.77-fold over infected control. The number of pods per plant in T9 was 8.8-fold higher than in the infected control, while yield per pot increased by 15.9-fold. Similarly, other two-microbe consortia significantly enhanced plant performance but were comparatively less effective than three- and four-microbe combinations.

Three-microbe consortia resulted in even greater improvements in plant growth and yield attributes. The best-performing three-microbe combination, *T. breve* 37F + *Pseudomonas* sp. 8B + *B. velezensis* 32B (T15), increased plant height by 1.49-fold, fresh weight by 2.22-fold and dry weight by 1.95-fold compared to infected control. The number of pods per plant in T15 treatment was 10.9-fold higher and yield per pot was 21.4-fold greater than in the infected control. The four-microbe consortium (T17: *T. breve* 37F + *Pseudomonas* sp. 8B + *A. aneurinilyticus* 16B + *B. velezensis* 32B) exhibited the highest improvements across all parameters. Compared to the infected control, T17 increased plant height by 1.66-fold, fresh weight by 2.81-fold and dry weight by 2.56-fold. The number of pods per plant increased 11.8-fold and the yield per pot was 21.4-fold higher than infected control (Table 5).

### 3.12 Validation of bioefficacy of microbial consortia against *Macrophomina phaseolina* of cluster bean under field condition

**Figure 5:**
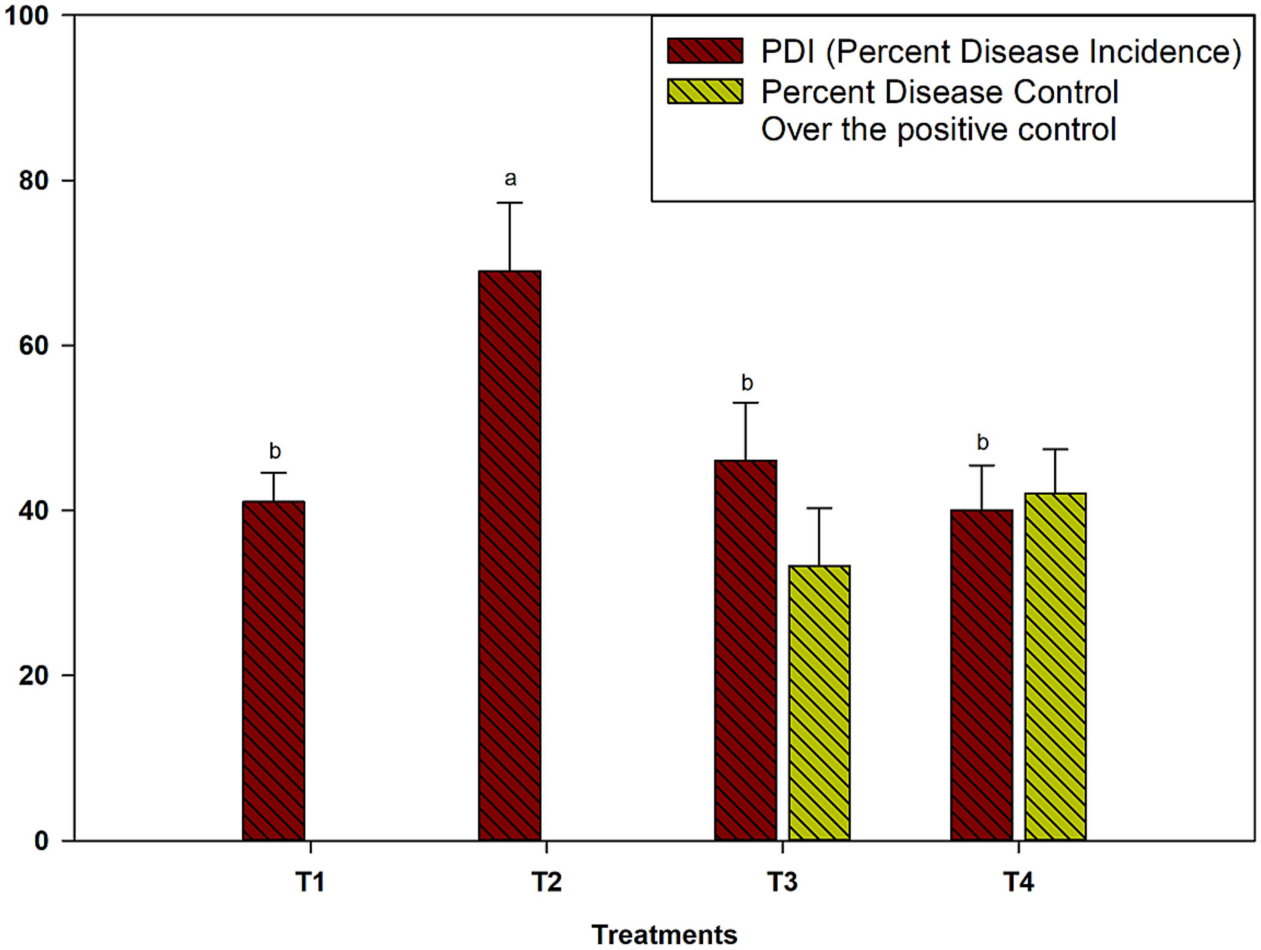
Validation of bioefficacy of microbial consortia against *M. phaseolina* of cluster bean under field condition during kharif season of 2024. Where, T1: No pathogen + No biocontrol agent (mock control); T2: Only *M. phaseolina* (infected control); T3: *Pseudomonas* sp. 8B + *A. aneurinilyticus* 16B + *Bacillus velezensis*32b + challenged with *M. phaseolina;* T4: *Pseudomonas* sp. 8B + *A. aneurinilyticus* 16B + *B. velezensis* 32B + *T. breve* 37F + challenged with *M. phaseolina*. #*Data are the average of five replicates ± SD; Grouping information between mean values of obtained data was carried out by Fisher LSD Method and 95% confidence (*p* ≤ 0.05). Different letter point out significant differences in a column.

The field validation of microbial consortia against *M. phaseolina* in cluster bean during the *kharif* season of 2024 revealed significant differences in disease suppression across treatments. The PDI varied significantly among treatments (*p* ≤ 0.05). The infected control exhibited the highest PDI (69.0%), indicating severe disease incidence (Figure 5). In contrast, the application of *Pseudomonas* sp. 8B + *A. aneurinilyticus* 16B + *B. velezensis* 32B (T3) significantly reduced the PDI to 46.0%, achieving a 33.3% reduction in disease severity compared to the infected control. Furthermore, the four-microbe consortium (*Pseudomonas* sp. 8B + *A. aneurinilyticus* 16B + *B. velezensis* 32B + *T. breve* 37F) was the most effective treatment, reducing the PDI to 40.0%, which corresponds to a 42.0% disease control over the infected control.

### 3.13 Effect of microbial consortia on clusterbean growth, biomass, yield and yield attributes against *Macrophomina phaseolina* of cluster bean under field condition

The field study demonstrated the significant impact of microbial consortia on cluster bean growth, biomass and yield under *M. phaseolina* infection. The infected control (T2) exhibited the lowest plant height, fresh weight, dry weight and yield. The statistical grouping confirmed that T3 (*Pseudomonas* sp. 8B + *A. aneurinilyticus* 16B + *Bacillus velezensis*32b) and T4 (*Pseudomonas* sp. 8B + *A. aneurinilyticus* 16B + *B. velezensis* 32B + *T. breve* 37F) significantly outperformed T2 (infected control), with T4 being superior in fresh weight, dry weight and yield. Compared to the infected control, T4 resulted in a 1.21-fold increase in plant height, 1.63-fold increase in fresh weight, 1.86-fold increase in dry weight and 2.79-fold increase in yield. Compared to the mock control (T1), T4 showed a 1.08-fold increase in plant height, 1.52-fold increase in fresh weight, 1.91-fold increase in dry weight and 1.67-fold increase in yield (Table 6).

**Table 6:** Effect of microbial consortia on clusterbean growth, biomass, yield and yield attributes against *Macrophomina phaseolina* of cluster bean under field condition during kharif season of 2024 year.

Treatments	Plant height (cm)	Fresh weight (g)	Dry weight (g)	Yield (kg/ha)
T1: No pathogen + No biocontrol agent (mock control)	135.0 ± 12.3^ab^	39.7 ± 11.5^bc^	6.8 ± 1.9^c^	227.5 ± 45.59^d^
T2: Only *Macrophomina phaseolina* (infected control)	121.2 ± 15.7^b^	37.0 ± 8.3^c^	7.0 ± 1.2^c^	136.6 ± 14.34^c^
T3: *Pseudomonas* sp. 8B + *Aneurinibacillus aneurinilyticus* 16B + *Bacillus velezensis* 32B + challenged with *M. phaseolina*	143.4 ± 7.0^a^	51.6 ± 7.4^ab^	10.4 ± 2.3^b^	334.0 ± 53.12^b^
T4: *Pseudomonas* sp. 8B + *Aneurinibacillus aneurinilyticus* 16B + *Bacillus velezensis* 32B + *Trichoderma breve* 37F + challenged with *M. phaseolina*	146.2 ± 12.1^a^	60.4 ± 11.5^a^	13.0 ± 1.6^a^	380.0 ± 18.12^a^

*Data are the average of five replicates ± SD; Grouping information between mean values of obtained data was carried out by Fisher LSD Method and 95% confidence (*P* ≤ 0.05). Different letter point out significant differences in a column.

## 4 Discussion

The screening of microbial isolates for antagonistic activity against *M. phaseolina* revealed a diverse range of fungal and bacterial strains with varying levels of inhibition, highlighting the importance of microbial diversity in biocontrol applications. Among the fungal isolates tested, *T. breve* 37F exhibited the highest inhibition rate, which corroborates earlier studies reporting *Trichoderma* species as potent biocontrol agents due to their ability to produce secondary metabolites, antibiotics and cell wall-degrading enzymes (Harman et al., 2004; Mukherjee et al., 2013). Similarly, among bacterial isolates, *Pseudomonas* sp. 8B, *A. aneurinilyticus* 16B and *B. velezensis* 32B showed significant antagonistic effects, supporting existing literature on the biocontrol efficacy of *Pseudomonas* and *Bacillus* species (Beneduzi et al., 2012; Asra et al., 2024).

The heat map analysis revealed clustering patterns based on functional traits, highlighting the co-occurrence of multiple biocontrol characteristics, including siderophore production, HCN, ammonia and chitinase. These traits have been widely studied and linked to the suppression of soil-borne pathogens (Compant et al., 2005; Bhattacharyya and Jha, 2012). Siderophore production by Pseudomonas and Bacillus isolates has been reported as a key mechanism for limiting iron availability to fungal pathogens, thereby inhibiting their growth (O’Sullivan and O’Gara, 1992). Additionally, HCN and ammonia production have been associated with microbial-induced plant defense responses and direct pathogen suppression (Sah et al., 2021).

The *in vitro* compatibility assay demonstrated a high degree of mutual compatibility between *T. breve* 37F and the bacterial isolates (*Pseudomonas* sp. 8B, *A. aneurinilyticus* 16B and *B. velezensis* 32B), indicating their potential as an effective microbial consortium. Previous studies have emphasized the importance of compatibility in microbial consortia for successful biocontrol applications (Singh and Das, 2024). The ability of these isolates to coexist without antagonistic interactions is crucial for formulation as biocontrol agents and for ensuring field efficacy (Berg et al., 2005). Morphological, physiological, and biochemical characterization further confirmed the identity and functional potential of these isolates. Observed variations in colony pigmentation, Gram reaction, and metabolic profiles align with existing taxonomic and functional studies of these microbial species (Logan, 2012). Moreover, the endospore-forming ability of *B. velezensis* 32B and *A. aneurinilyticus* 16B suggests resilience under field conditions, a desirable trait for biocontrol formulations (Shafi et al., 2017).

The pot experiment evaluating the bioefficacy of selected biocontrol agents against *M. phaseolina* demonstrated that consortium treatments were significantly more effective in suppressing disease severity compared to individual strains. This finding aligns with previous studies emphasizing the importance of microbial compatibility in enhancing biocontrol efficacy (Minchev et al., 2021; Solórzano et al., 2025). Microbial consortia often exhibit synergistic interactions, where multiple mechanisms, including competition, antibiosis and ISR, contribute to improved pathogen suppression (Mendes et al., 2011). Studies by Lugtenberg and Kamilova (2009) and Akhtar and Siddiqui (2010) have demonstrated that microbial consortia can enhance disease resistance by modulating plant hormonal pathways and inducing systemic resistance, further supporting the observed efficacy in this study.

Present results are also consistent with studies by Vinale et al. (2008), which demonstrated that microbial consortia exhibit enhanced plant protection by simultaneously inhibiting pathogen growth and promoting plant defense responses. The lower efficacy of individual treatments suggests that a single biocontrol agent may lack the broad-spectrum activity required for effective disease suppression. Previous research by Howell (2003) and Shoresh et al. (2010) has highlighted that while *Trichoderma* species are potent biocontrol agents, their effectiveness is enhanced when combined with other beneficial microbes that contribute additional mechanisms, such as nutrient competition and ISR activation.

In present study, microbial consortia significantly enhanced chlorophyll and carotenoid content in cluster bean, contributing to improved plant health and stress tolerance against *M. phaseolina*. The highest values for chlorophyll a, chlorophyll b and total chlorophyll were observed in the four-microbe consortium treatment, demonstrating a significant increase over both the infected and mock controls. The increased photosynthetic pigment content in consortial treatments suggests improved photosynthetic efficiency, likely due to reduced pathogen-induced stress and enhanced nutrient uptake facilitated by beneficial microbes. The improved chlorophyll and carotenoid content in plants treated with microbial consortia can be attributed to multiple factors, including ISR, improved nutrient acquisition, and reduced oxidative stress. *Trichoderma* species have been reported to enhance plant growth by producing phytohormones and solubilizing nutrients. For instance, Lei and Zhang (2015) reported that *T. asperellum* Q1 enhances phosphorus availability by solubilizing phosphate and producing phytohormones, improving cucumber growth under salt stress. Additionally, Trichoderma-derived bioactive metabolites enhance nutrient uptake and adaptation under stress (Contreras-Cornejo et al., 2024).

The enhanced accumulation of phenolic compounds, flavonoids, tannins and increased antioxidant activity in plants treated with microbial consortia underscores the efficacy of such treatments in bolstering plant defenses against *M. phaseolina.* These biochemical responses play pivotal roles in reinforcing plant resilience against pathogenic attacks. Phenolic compounds and flavonoids are integral to plant defense mechanisms due to their antimicrobial properties and role in signaling pathways associated with ISR. Their accumulation can inhibit pathogen proliferation and fortify plant structural barriers. Studies have demonstrated that higher concentrations of these compounds correlate with enhanced resistance to pathogens. For instance, research indicates that secondary metabolites, including phenolics, possess antifungal characteristics effective against pathogens like *M. phaseolina* (Marquez et al., 2021). Tannins contribute to plant defense by complexing with proteins, thereby reducing pathogen virulence. The significant increase in tannin content observed in consortial treatments suggests a robust defensive response. While specific studies on tannin accumulation in response to *M. phaseolina* are limited, the general role of tannins in plant defense is well-documented. For example, certain plant extracts rich in tannins have exhibited antifungal activity against soil-borne pathogens (Bobbarala et al., 2009).

In present study, enhanced antioxidant activity indicates a plant’s improved capability to mitigate oxidative stress induced by pathogen invasion. Antioxidants neutralize reactive oxygen species, thereby protecting cellular integrity. Studies have shown that plant extracts with elevated antioxidant properties can effectively suppress fungal pathogens, including *M. phaseolina* (Dilkin et al., 2022). The observed reduction in electrolyte leakage in plants treated with microbial consortia indicates enhanced membrane stability and a strengthened defense response against *M. phaseolina* infection. Electrolyte leakage is a common indicator of cell membrane integrity loss due to various stresses, including pathogen attacks. Demidchik et al. (2014) reported that stress-induced electrolyte leakage is primarily associated with potassium (K^+^) efflux from plant cells, mediated by plasma membrane cation channels. This K^+^ efflux can lead to programmed cell death (PCD) under severe stress conditions. The application of beneficial microbial consortia has been shown to mitigate such stress responses.

A study Minchev et al. (2021) highlighted that microbial consortia, composed of beneficial bacteria (*Bacillus amyloliquefaciens* strains CECT 8238 and CECT 8237, *Pseudomonas chlororaphis* MA 342 and *Pseudomonas azotoformans* F30A) and fungi (*Trichoderma harzianum* strains T22 and ESALQ1306), can effectively control both root and foliar diseases in tomato plants. These consortia not only suppress pathogen growth but also enhance the plant’s innate defense mechanisms, leading to improved plant health and reduced oxidative stress.

In present study, the significant increase in enzymatic activities such as POX, PPO, PAL, TAL, SOD and CAT in plants treated with biocontrol consortia suggests a strong activation of plant defense mechanisms. These findings are aligned with Sharma et al. (2018) which demonstrated that a PGPR consortium of *Pseudomonas putida* CRN-09 and *Bacillus subtilis* CRN-16 induced systemic resistance in mung bean against *M. phaseolina* by enhancing key defense enzymes such as POX, PPO, phenylalanine ammonia PAL, β-1,3-glucanase, and chitinase. The upregulation of similar enzymatic pathways in our study further supports the hypothesis that ISR activation by biocontrol agents is a conserved defense mechanism. POX and PPO are pivotal enzymes in plant defense mechanisms. POX is integral to lignin biosynthesis, facilitating the oxidative polymerization of monolignols into lignin, thereby reinforcing cell walls against pathogen intrusion. Similarly, PPO catalyzes the oxidation of phenolic compounds to quinones, which subsequently polymerize, contributing to cell wall fortification and exhibiting antimicrobial properties (Herrero et al., 2013; Aquino-Bolaños and Mercado-Silva, 2004). The observed induction of POX and PPO activities in consortium treatments underscores the synergistic effect of multiple biocontrol agents in enhancing host defenses. This enzymatic upregulation leads to strengthened cell walls and increased antimicrobial compound production, impeding pathogen progression.

PAL and TAL are key enzymes in the phenylpropanoid pathway, leading to the synthesis of phenolics, flavonoids and phytoalexins, which serve as antimicrobial compounds (Kong, 2015; Sharma et al., 2022). The higher PAL and TAL activities in consortial treatments indicate an upregulation of secondary metabolite biosynthesis, which strengthens plant immunity against *M. phaseolina*. Imran et al. (2023) reported that the application of *Trichoderma* culture filtrates (*T. harzianum, T. longibrachiatum*, and *T. atroviride*) significantly enhanced enzymatic activities, including phenylalanine ammonia-lyase (PAL), peroxidase (POD) and polyphenol oxidase (PPO) in tomato. This led to increased antioxidant defense and reduced disease severity against *Alternaria solani* under greenhouse and open field conditions.

In present study, pathogen invasion led to excessive ROS generation, causing oxidative stress and cellular damage. The significant enhancement of SOD, CAT and POX activities in biocontrol-treated plants suggests a reinforced ROS-scavenging mechanism, thereby mitigating oxidative damage. SOD catalyzes the conversion of superoxide radicals into hydrogen peroxide (H₂O₂), which is subsequently detoxified by CAT and POX to prevent oxidative stress (Weydert and Cullen, 2010). The significant reduction in peroxide and superoxide accumulation, as evidenced by histochemical staining, further corroborates the protective role of microbial consortia in maintaining redox homeostasis (Imran et al., 2023).

Our findings demonstrated that both individual and consortia-based microbial applications led to marked improvements in plant health, with multi-microbe consortia outperforming single-agent treatments. These findings are consistent with previous research highlighting the benefits of microbial consortia in plant disease management and growth promotion (Thakkar and Saraf, 2015; Sharma et al., 2025). Our findings align with the study of Kapadiya et al. (2023) which demonstrated that microbial consortia of *Trichoderma* and *Pseudomonas fluorescens* effectively reduced stem and root rot disease in okra while enhancing yield. Sharma et al. (2018) reported the synergistic effects of PGPR consortia (*Pseudomonas putida* CRN-09 and *Bacillus subtilis* CRN-16) in suppressing *M. phaseolina* causes dry root of mung bean, similar to our findings where a four-microbe consortium (*T. breve* 37F, *Pseudomonas* sp. 8B, *A. aneurinilyticus* 16B, *B. velezensis* 32B) provided the highest disease suppression and yield enhancement in cluster bean under both pot and field conditions.

Sulieman et al. (2025) reported that carefully selected and compatible microbial consortia not only suppress pathogenic infections but also enhance plant growth and resilience by activating systemic defense mechanisms. Present findings are aligned with Charpe et al. (2025) as both studies demonstrate that compatible microbial consortia, including *Trichoderma*, *Bacillus* and *Pseudomonas* species, can induce systemic resistance in plants, enhance physiological and biochemical defense responses and improve growth and yield. Moreover, present work complements their emphasis on translating lab-level observations into field-level efficacy by validating the four-microbe consortium under arid field conditions, highlighting the practical potential of microbial-mediated induced resistance for sustainable crop protection. Our findings are also corroborated with Kumar et al. (2021), which reported that a PGPR consortium consisting of *Pseudomonas* sp., *Azotobacter* sp., and *Bacillus* sp. significantly enhanced plant growth, biomass, and yield in tomato by improving root and shoot development, nutrient uptake, and pathogen suppression.

## 5 Conclusion

This study established that *T. breve* 37F, *Pseudomonas* sp. 8B, *A. aneurinilyticus* 16B and *B. velezensis* 32B are potent biocontrol agents against *M. phaseolina*, combining strong antagonistic activity, plant growth-promoting traits and mutual compatibility. Among them, the four-microbe consortium proved most effective, significantly reducing disease severity while enhancing plant growth, biomass and yield in both controlled pot experiments and field conditions. The consortium’s benefits were associated with improved physiological and biochemical responses, including enhanced chlorophyll content, accumulation of phenolic compounds, activation of antioxidant defense enzymes and reduction of oxidative stress and electrolyte leakage. These findings demonstrate the consortium’s holistic impact on plant health, bridging laboratory results with practical field-level performance. Overall, the study highlights microbial consortia as a sustainable, eco-friendly strategy for managing dry root rot in cluster bean under arid conditions. Future research should focus on large-scale field validation, development of stable bioformulations, integration into sustainable cropping systems, and farmer-oriented applications to maximize the agricultural benefits of this biocontrol approach.

## Data Availability

The datasets presented in this study can be found in online repositories. The names of the repository/repositories and accession number(s) can be found in the article/Supplementary material.

## Supplementary material

Supplementary Figure 1 Pot experiment for evaluation of bioefficacy of promising biocontrol agents in individual and consortium mode against *M. phaseolina* causing dry root rot of cluster bean.

Supplementary Figure 2 Field experiment for evaluation of bioefficacy of promising microbial consortia against *M. phaseolina* causing dry root rot of cluster bean.

Supplementary Figure 3 *In vitro* antagonistic assay of fungal isolates against *M. phaseolina*.

Supplementary Figure 4 *In vitro* antagonistic assay of bacterial isolates against *M. phaseolina*.
