# Supplementary figures and images for: Harnessing microbial consortia for induced systemic resistance and sustainable management of dry root rot in cluster bean under hot arid climatic conditions
Source: Front Microbiol. 2025 Oct 22;16:1699101. doi: 10.3389/fmicb.2025.1699101 (PMC12586090; doi:10.3389/fmicb.2025.1699101)

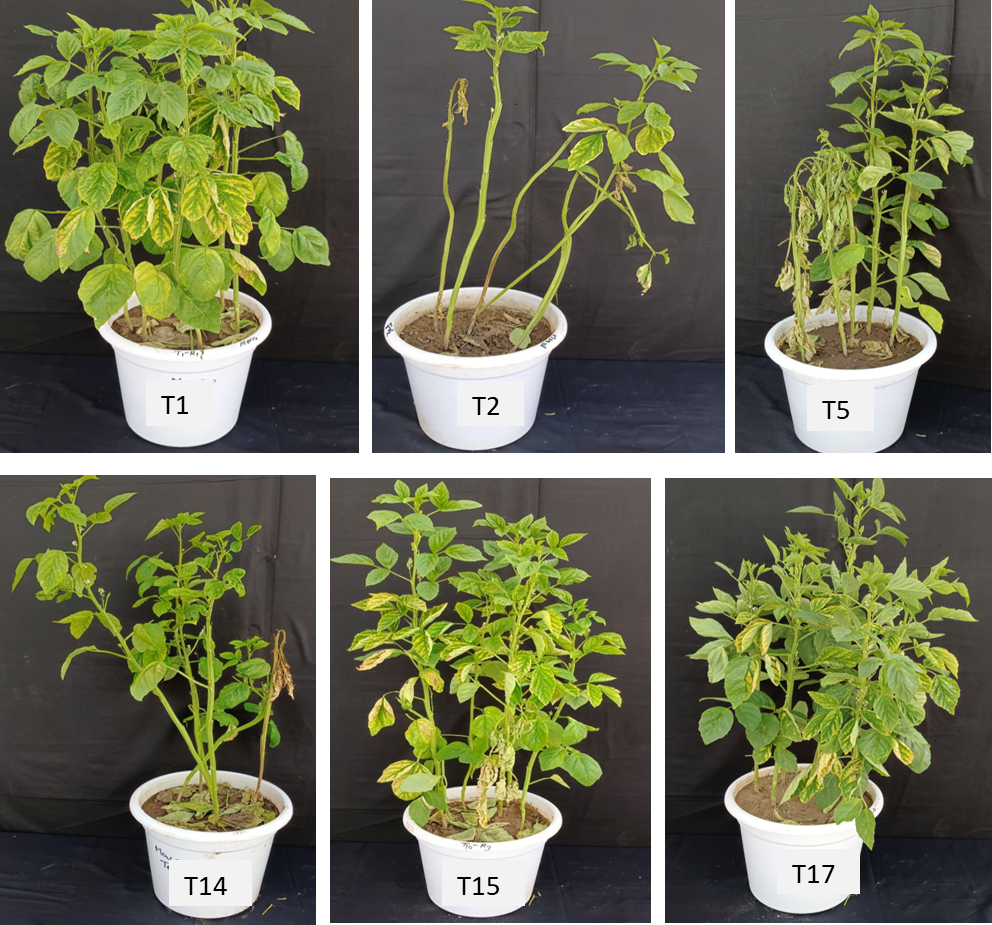

Supplement: Supplementary Figure 1 — Pot experiment for evaluation of bioefficacy of promising biocontrol agents in individual and consortium mode against M. phaseolina causing dry root rot of cluster bean. [file Image_1.tif]

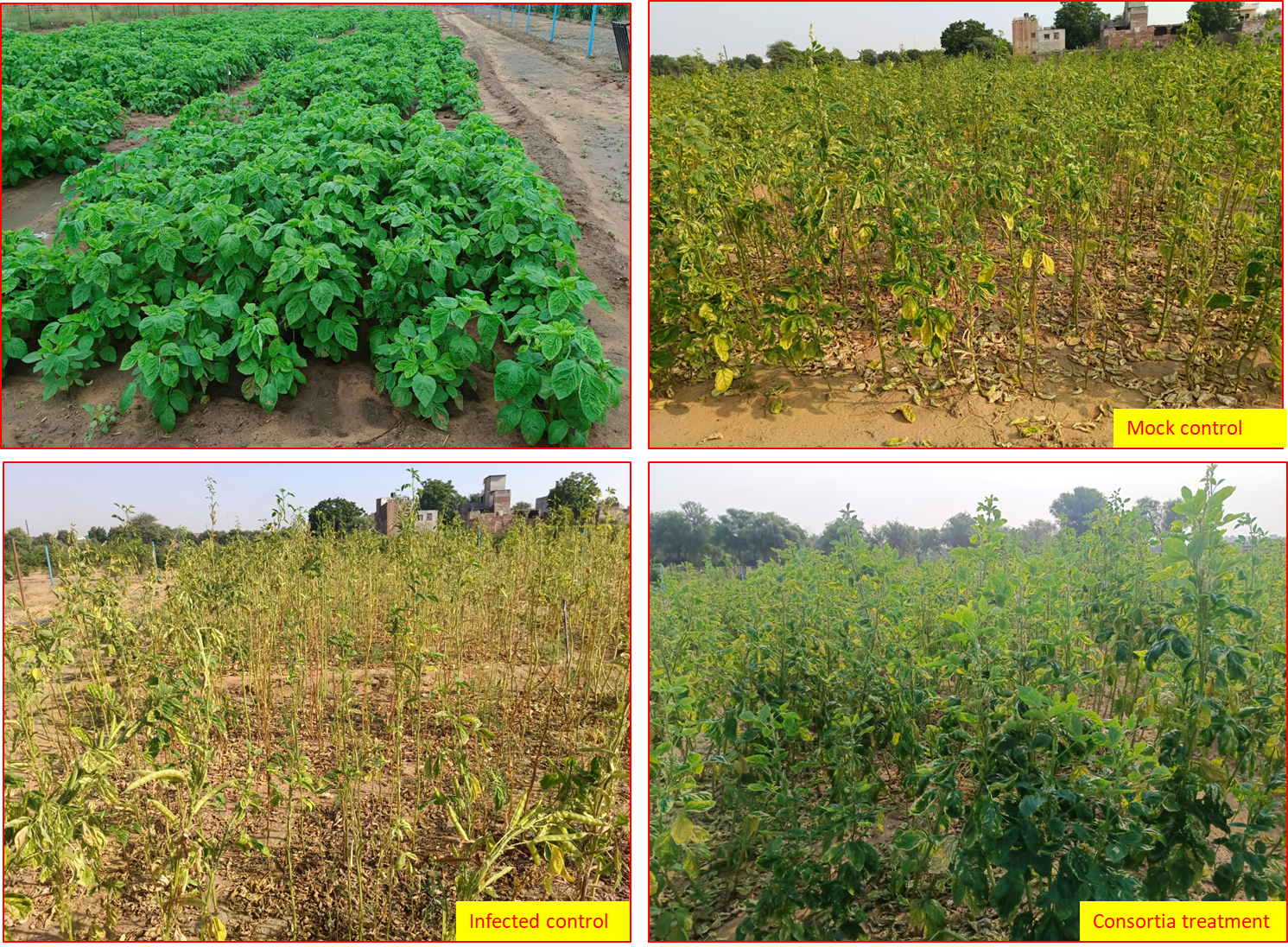

Supplement: Supplementary Figure 2 — Field experiment for evaluation of bioefficacy of promising microbial consortia against M. phaseolina causing dry root rot of cluster bean. [file Image_2.tif]

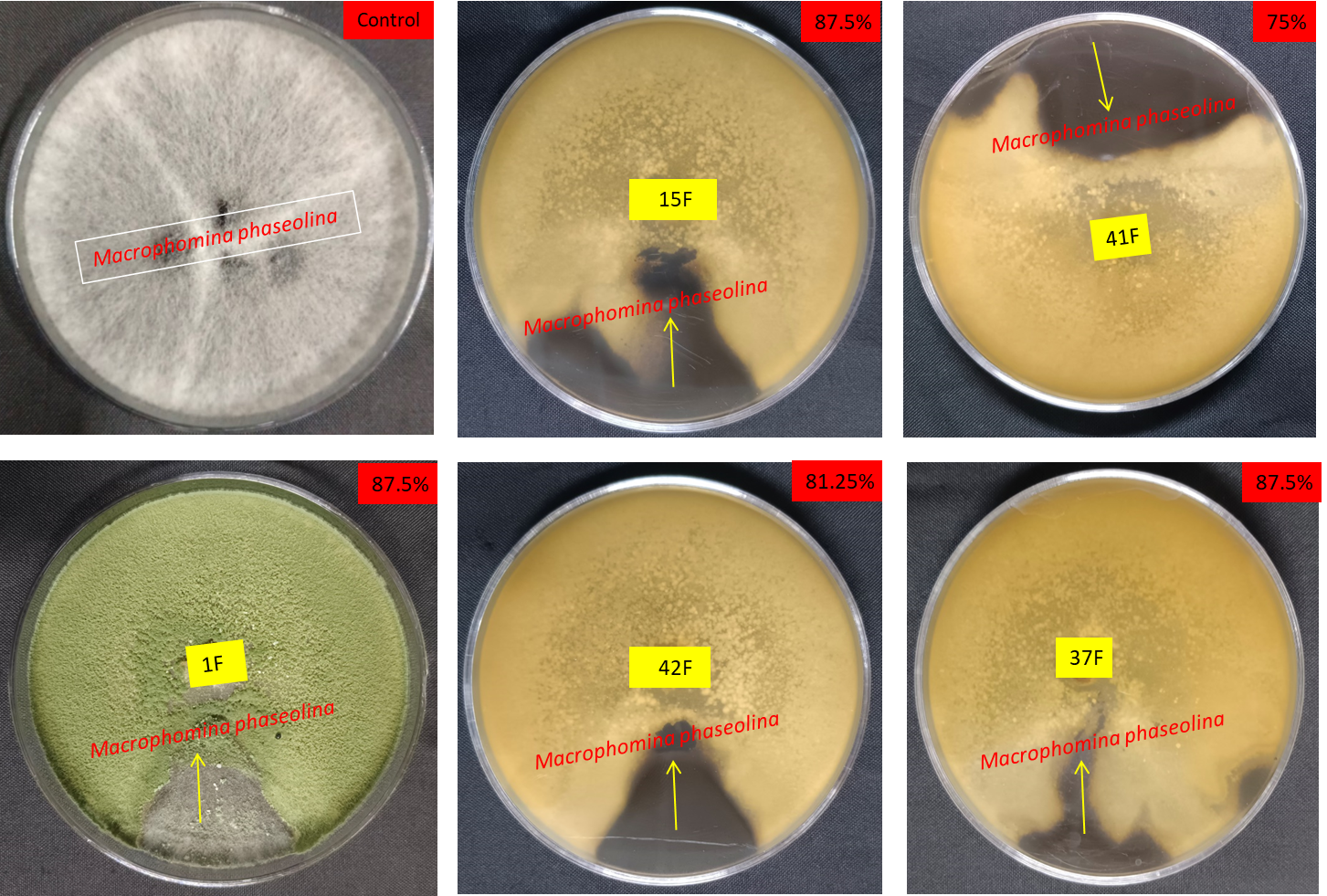

Supplement: Supplementary Figure 3 — In vitro antagonistic assay of fungal isolates against M. phaseolina. [file Image_3.tif]

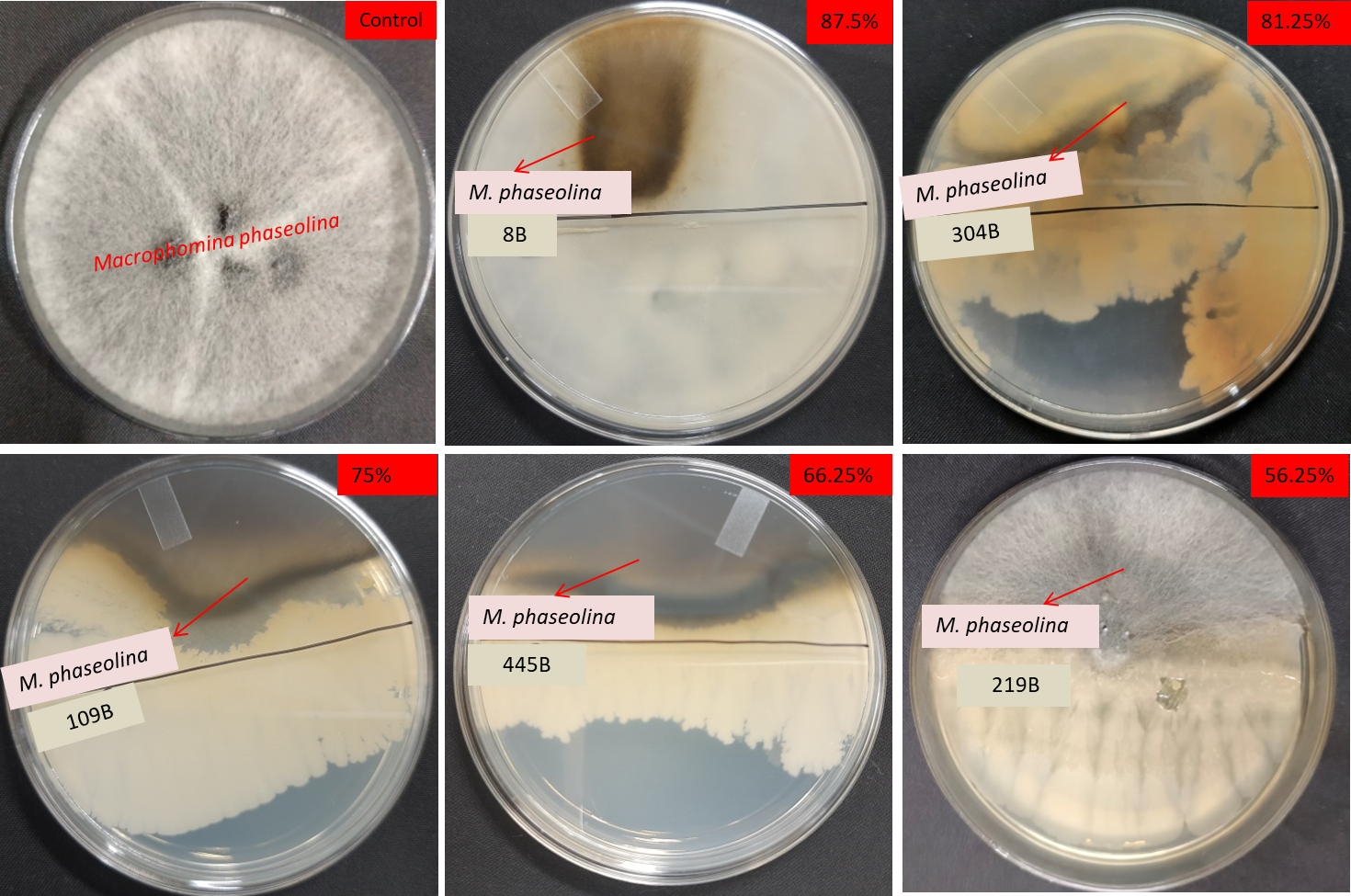

Supplement: Supplementary Figure 4 — In vitro antagonistic assay of bacterial isolates against M. phaseolina. [file Image_4.tif]
