# Supplementary material for: Harnessing microbial consortia for induced systemic resistance and sustainable management of dry root rot in cluster bean under hot arid climatic conditions
Source: Front Microbiol. 2025 Oct 22;16:1699101. doi: 10.3389/fmicb.2025.1699101 (PMC12586090; doi:10.3389/fmicb.2025.1699101)
Supplement: Supplementary file 5 [file Table_1.docx]

**Supplementary table 1 *In vitro* antagonistic assay of fungal isolates against *Macrophomina phaseolina***

| S.N. | Dual test | Diameter of T (cm) | Diameter of C | %Inhibition |
| --- | --- | --- | --- | --- |
| 1 | *Macrophomina phaseolina*X 1F | 1 | 8 | 87.5 |
| 2 | *Macrophomina phaseolina*X 2F | 1 | 8 | 87.5 |
| 3 | *Macrophomina phaseolina*X 3F | 1 | 8 | 87.5 |
| 4 | *Macrophomina phaseolina*X 4F | 1 | 8 | 87.5 |
| 5 | *Macrophomina phaseolina*X 5F | 3 | 8 | 62.5 |
| 6 | *Macrophomina phaseolina*X 6F | 1 | 8 | 87.5 |
| 7 | *Macrophomina phaseolina*X 7F | 1 | 8 | 87.5 |
| 8 | *Macrophomina phaseolina*X 8F | 1 | 8 | 87.5 |
| 9 | *Macrophomina phaseolina*X 9F | 1 | 8 | 87.5 |
| 10 | *Macrophomina phaseolina*X 10F | 1 | 8 | 87.5 |
| 11 | *Macrophomina phaseolina*X 11F | 1 | 8 | 87.5 |
| 12 | *Macrophomina phaseolina*X 12F | 1 | 8 | 87.5 |
| 13 | *Macrophomina phaseolina*X 13F | 1 | 8 | 87.5 |
| 14 | *Macrophomina phaseolina*X 14F | 1 | 8 | 87.5 |
| 15 | *Macrophomina phaseolina*X 15F | 1 | 8 | 87.5 |
| 16 | *Macrophomina phaseolina*X 16F | 6 | 8 | 25 |
| 17 | *Macrophomina phaseolina*X 17F | 6 | 8 | 25 |
| 18 | *Macrophomina phaseolina*X 18F | 6 | 8 | 25 |
| 19 | *Macrophomina phaseolina*X 19F | 2 | 8 | 75 |
| 20 | *Macrophomina phaseolina*X 20F | 4 | 8 | 50 |
| 21 | *Macrophomina phaseolina*X 21F | 3 | 8 | 62.5 |
| 22 | *Macrophomina phaseolina*X 22F | 7.4 | 8 | 7.5 |
| 23 | *Macrophomina phaseolina*X 23F | 7.3 | 8 | 8.75 |
| 24 | *Macrophomina phaseolina*X 24F | 7 | 8 | 12.5 |
| 25 | *Macrophomina phaseolina*X 25F | 7 | 8 | 12.5 |
| 26 | *Macrophomina phaseolina*X 26F | 7 | 8 | 12.5 |
| 27 | *Macrophomina phaseolina*X 27F | 7 | 8 | 12.5 |
| 28 | *Macrophomina phaseolina*X 28F | 7 | 8 | 12.5 |
| 29 | *Macrophomina phaseolina*X 37F | 1 | 8 | 87.5 |
| 30 | *Macrophomina phaseolina*X 38F | 6.5 | 8 | 18.75 |
| 31 | *Macrophomina phaseolina*X 39F | 6 | 8 | 25 |
| 32 | *Macrophomina phaseolina*X 40F | 4.2 | 8 | 47.5 |
| 33 | *Macrophomina phaseolina*X 41F | 2 | 8 | 75 |
| 34 | *Macrophomina phaseolina*X 42F | 1.5 | 8 | 81.25 |
| 35 | *Macrophomina phaseolina*X 43F | 1 | 8 | 87.5 |
| 36 | *Macrophomina phaseolina*X 44F | 1 | 8 | 87.5 |

**Supplementary table 2 *In vitro* antagonistic assay of bacterial isolates against *Macrophomina phaseolina***

| S.No. | Dual test | Diameter of T | Diameter of C | %Inhibition |
| --- | --- | --- | --- | --- |
| 1 | *Macrophomina phaseolina X 2B* | 2 | 8 | 75 |
| 2 | *Macrophomina phaseolina X 3B* | 3.5 | 8 | 56.25 |
| 3 | *Macrophomina phaseolina X 5B* | 2.8 | 8 | 65 |
| 4 | *Macrophomina phaseolina X 7B* | 3.5 | 8 | 56.25 |
| 5 | *Macrophomina phaseolina X 8B* | 1 | 8 | 87.5 |
| 6 | *Macrophomina phaseolina X 10B* | 1 | 8 | 87.5 |
| 7 | *Macrophomina phaseolina X 12B* | 3.5 | 8 | 56.25 |
| 8 | *Macrophomina phaseolina X 14B* | 2 | 8 | 75 |
| 9 | *Macrophomina phaseolina X 16B* | 1 | 8 | 87.5 |
| 10 | *Macrophomina phaseolina X 17B* | 1.8 | 8 | 77.5 |
| 11 | *Macrophomina phaseolina X 20B* | 1 | 8 | 87.5 |
| 12 | *Macrophomina phaseolina X 26B* | 2.7 | 8 | 66.25 |
| 13 | *Macrophomina phaseolina X 28B* | 3 | 8 | 62.5 |
| 14 | *Macrophomina phaseolina X 29B* | 3.2 | 8 | 60 |
| 15 | *Macrophomina phaseolina X 30B* | 3 | 8 | 62.5 |
| 16 | *Macrophomina phaseolina X31B* | 2 | 8 | 75 |
| 17 | *Macrophomina phaseolina X 32B* | 1 | 8 | 87.5 |
| 18 | *Macrophomina phaseolina X 34B* | 1 | 8 | 87.5 |
| 19 | *Macrophomina phaseolina X 40B* | 3 | 8 | 62.5 |
| 20 | *Macrophomina phaseolina X 48B* | 1 | 8 | 87.5 |
| 21 | *Macrophomina phaseolina X 68B* | 3.1 | 8 | 61.25 |
| 22 | *Macrophomina phaseolina X 72B* | 1 | 8 | 87.5 |
| 23 | *Macrophomina phaseolina X 78B* | 3.8 | 8 | 52.5 |
| 24 | *Macrophomina phaseolina X 89B* | 3.5 | 8 | 56.25 |
| 25 | *Macrophomina phaseolina X 97B* | 3 | 8 | 62.5 |
| 26 | *Macrophomina phaseolina X 98B* | 3 | 8 | 62.5 |
| 27 | *Macrophomina phaseolina X 109B* | 2 | 8 | 75 |
| 28 | *Macrophomina phaseolina X 131B* | 3.5 | 8 | 56.25 |
| 29 | *Macrophomina phaseolina X 169B* | 1 | 8 | 87.5 |
| 30 | *Macrophomina phaseolina X 179B* | 2.8 | 8 | 65 |
| 31 | *Macrophomina phaseolina X 192B* | 3.9 | 8 | 51.25 |
| 32 | *Macrophomina phaseolina X 195B* | 2.2 | 8 | 72.5 |
| 33 | *Macrophomina phaseolina X 197B* | 1.8 | 8 | 77.5 |
| 34 | *Macrophomina phaseolina X 200B* | 2.6 | 8 | 67.5 |
| 35 | *Macrophomina phaseolina X 201B* | 3 | 8 | 62.5 |
| 36 | *Macrophomina phaseolina X 202B* | 3.5 | 8 | 56.25 |
| 37 | *Macrophomina phaseolina X 207B* | 3.2 | 8 | 60 |
| 38 | *Macrophomina phaseolina X 209B* | 3 | 8 | 62.5 |
| 39 | *Macrophomina phaseolina X 211B* | 3.1 | 8 | 61.25 |
| 40 | *Macrophomina phaseolina X 215B* | 2.4 | 8 | 70 |
| 41 | *Macrophomina phaseolina X 216B* | 2.4 | 8 | 70 |
| 42 | *Macrophomina phaseolina X 217B* | 2 | 8 | 75 |
| 43 | *Macrophomina phaseolina X 219B* | 3.5 | 8 | 56.25 |
| 44 | *Macrophomina phaseolina X 220B* | 3.5 | 8 | 56.25 |
| 45 | *Macrophomina phaseolina X 221B* | 3.5 | 8 | 56.25 |
| 46 | *Macrophomina phaseolina X 223B* | 2.5 | 8 | 68.75 |
| 47 | *Macrophomina phaseolina X 236B* | 2.5 | 8 | 68.75 |
| 48 | *Macrophomina phaseolina X 242B* | 3.5 | 8 | 56.25 |
| 49 | *Macrophomina phaseolina X 248B* | 1 | 8 | 87.5 |
| 50 | *Macrophomina phaseolina X 257B* | 2 | 8 | 75 |
| 51 | *Macrophomina phaseolina X 261B* | 2.3 | 8 | 71.25 |
| 52 | *Macrophomina phaseolina X 267B* | 4.8 | 8 | 40 |
| 53 | *Macrophomina phaseolina X 271B* | 1.7 | 8 | 78.75 |
| 54 | *Macrophomina phaseolina X 278B* | 2 | 8 | 75 |
| 55 | *Macrophomina phaseolina X 290B* | 2 | 8 | 75 |
| 56 | *Macrophomina phaseolina X 295B* | 3.5 | 8 | 56.25 |
| 57 | *Macrophomina phaseolina X 301B* | 1 | 8 | 87.5 |
| 58 | *Macrophomina phaseolina X 302B* | 1 | 8 | 87.5 |
| 59 | *Macrophomina phaseolina X 304B* | 1.5 | 8 | 81.25 |
| 60 | *Macrophomina phaseolina X 310B* | 3.5 | 8 | 56.25 |
| 61 | *Macrophomina phaseolina X 325B* | 3.8 | 8 | 52.5 |
| 62 | *Macrophomina phaseolina X 340B* | 3.5 | 8 | 56.25 |
| 63 | *Macrophomina phaseolina X 346B* | 2.5 | 8 | 68.75 |
| 64 | *Macrophomina phaseolina X 356B* | 2.5 | 8 | 68.75 |
| 65 | *Macrophomina phaseolina X 390B* | 2.5 | 8 | 68.75 |
| 66 | *Macrophomina phaseolina X 391B* | 2 | 8 | 75 |
| 67 | *Macrophomina phaseolina X 415B* | 2.5 | 8 | 68.75 |
| 68 | *Macrophomina phaseolina X 444B* | 3 | 8 | 62.5 |
| 69 | *Macrophomina phaseolina X 445B* | 2.7 | 8 | 66.25 |
| 70 | *Macrophomina phaseolina X 471B* | 3.5 | 8 | 56.25 |
| 71 | *Macrophomina phaseolina X 475B* | 3 | 8 | 62.5 |
| 72 | *Macrophomina phaseolina X 476B* | 3 | 8 | 62.5 |
| 73 | *Macrophomina phaseolina X 477B* | 5 | 8 | 37.5 |

**Supplementary Table 3** Morphological characterization of potent biocontrol bacterial isolates

| **S.No.** | **Colony morphology** | **Bacterial Isolates** | | |
| --- | --- | --- | --- | --- |
|  |  | 8B | 16B | 32B |
| 1 | Size | medium | medium | medium |
| 2 | Shape | circular with a smooth, well-defined edge | circular with a well-defined edge | Circular with smooth or slightly irregular edges |
| 3 | Margin | entire (smooth, not serrated). | entire (smooth, without irregularities) | entire to slightly undulate |
| 4 | Opacity | translucent to opaque | opaque | opaque |
| 5 | Elevation | flat or slightly raised | raised or convex | raised to convex |
| 6 | Texture | smooth or mucoid (sticky), | smooth or slightly rough surface | mucoid |
| 7 | Pigmentation | Greenish yellow | white | white |
|  | **Cell morphology** | | | |
| 8 | Gram reaction | Gram-negative | Gram-positive | Gram-positive |
| 9 | Shape | rod | rod | rod |
| 10 | Endospore formation | - | + | + |

**Supplementary Table 4** Microscopic and macroscopic characteristics of *Trichoderma breve*37F

| **Sl.N.** | **Characteristics** | **Fungal isolate** |
| --- | --- | --- |
|  |  | **37F** |
| 1. | Spore shape | ellipsoidal or globose |
| 2. | Colour | colonies appear white initially and develop green pigmentation as they mature |
| 3. | Spore arrangement | Conidia are produced on phialides, which are flask-shaped structures. These phialidesarranged in clusters on branched conidiophores. |
| 4. | Hyphae | Septate, hyaline, and smooth |
| 5. | Fruiting bodies | Conidiophores branched with flask-shaped phialides |
| 6. | **Colony Appearance** | Colonies are typically fast-growing and cover anpotato dextrose agar (PDA) plates within a week when incubated at 25–30°C. On PDA, colonies appear white initially and develop green or yellow pigmentation as they mature. |

**Supplementary Table 5** Physiological and biochemical characterization of potent biocontrol bacterial isolates

| **S.No.** | **Test/Characteristic** | **8B** | **16B** | **32B** |
| --- | --- | --- | --- | --- |
| **1** | **Lactose** | Positive | Negative | Negative |
| **2** | **Xylose** | Positive | Positive | Positive |
| **3** | **Maltose** | Positive | Positive | Positive |
| **4** | **Fructose** | Positive | Positive | Positive |
| **5** | **Dextrose (Glucose)** | Positive | Positive | Positive |
| **6** | **Galactose** | Positive | Positive | Positive |
| **7** | **Raffinose** | Negative | Negative | Negative |
| **8** | **Trehalose** | Positive | Positive | Positive |
| **9** | **Melibiose** | Positive | Negative | Negative |
| **10** | **Sucrose** | Positive | Positive | Positive |
| **11** | **L-Arabinose** | Positive | Positive | Positive |
| **12** | **Mannose** | Positive | Positive | Positive |
| **13** | **Inulin** | Negative | Negative | Negative |
| **14** | **Sodium gluconate** | Positive | Positive | Positive |
| **15** | **Glycerol** | Positive | Positive | Positive |
| **16** | **Salicin** | Negative | Positive | Positive |
| **17** | **Dulcitol** | Negative | Negative | Negative |
| **18** | **Inositol** | Negative | Negative | Negative |
| **19** | **Sorbitol** | Negative | Positive | Positive |
| **20** | **Mannitol** | Positive | Positive | Positive |
| **21** | **Adonitol** | Negative | Negative | Negative |
| **22** | **Arabitol** | Negative | Negative | Negative |
| **23** | **Erythritol** | Negative | Negative | Negative |
| **24** | **α-Methyl-D-glucoside** | Negative | Negative | Negative |
| **25** | **Rhamnose** | Positive | Positive | Positive |
| **26** | **Cellobiose** | Negative | Positive | Positive |
| **27** | **Melezitose** | Negative | Negative | Negative |
| **28** | **α-Methyl-D-mannoside** | Negative | Negative | Negative |
| **29** | **Xylitol** | Negative | Negative | Negative |
| **30** | **D-Arabinose** | Negative | Negative | Negative |
| **31** | **Sorbose** | Negative | Negative | Negative |
| **32** | **Citrate Utilization** | Positive | Positive | Positive |
| **33** | **Malonate Utilization** | Negative | Negative | Negative |
| **34** | **ONPG** | Negative | Negative | Negative |
| **35** | **Esculin Hydrolysis** | Negative | Positive | Positive |
| **36** | **Lysine Utilization** | Negative | Positive | Positive |
| **37** | **Ornithine Utilization** | Negative | Positive | Positive |
| **38** | **Urease** | Positive | Negative | Negative |
| **39** | **Phenylalanine Deamination** | Negative | Negative | Negative |
| **40** | **Nitrate Reductase** | Positive | Positive | Positive |
| **41** | **H₂S Production** | Negative | Negative | Negative |
| **42** | **Oxidase** | Positive | Positive | Positive |
| **43** | **Casein Hydrolysis** | Positive | Positive | Positive |
| **44** | **Catalase Test** | Positive | Positive | Positive |
| **45** | **KOH Test** | Positive | Negative | Negative |
